# Ancestral sequences from an elite neutralizer proximal to the development of neutralization resistance as a potential source of HIV vaccine immunogens

**DOI:** 10.1371/journal.pone.0213409

**Published:** 2019-04-10

**Authors:** Kathryn A. Mesa, Bin Yu, Terri Wrin, Christos J. Petropoulos, Grant H. Pogson, David L. Alexander, Gerardo Perez, Sara M. O’Rourke, Faruk Sinangil, Joseph Robinson, Marcus A. Conant, Phillip W. Berman

**Affiliations:** 1 Department of Biomolecular Engineering, Baskin School of Engineering, University of California Santa Cruz, Santa Cruz, CA, United States of America; 2 Monogram Biosciences, South San Francisco, CA, United States of America; 3 Department of Ecology and Evolutionary Biology, University of California Santa Cruz, Santa Cruz, CA, United States of America; 4 Global Solutions for Infectious Diseases, South San Francisco, California, United States of America; 5 The Conant Medical Foundation, San Francisco, California, United States of America; Macfarlane Burnet Institute for Medical Research and Public Health, Australia, AUSTRALIA

## Abstract

A major challenge in HIV vaccine development is the identification of immunogens able to elicit broadly neutralizing antibodies (bNAbs). While remarkable progress has been made in the isolation and characterization of bNAbs, the epitopes they recognize appear to be poorly immunogenic. Thus, none of the candidate vaccines developed to date has induced satisfactory levels of neutralizing antibodies to the HIV envelope protein (Env). One approach to the problem of poor immunogenicity is to build vaccines based on envelope (*env*) genes retrieved from rare individuals termed elite neutralizers (ENs) who at one time possessed specific sequences that stimulated the formation of bNAbs. Env proteins selected from these individuals could possess uncommon, yet to be defined, structural features that enhance the immunogenicity of epitopes recognized by bNAbs. Here we describe the recovery of *env*s from an EN that developed unusually broad and potent bNAbs. As longitudinal specimens were not available, we combined plasma and provirus sequences acquired from a single time-point to infer a phylogenetic tree. Combining ancestral reconstruction data with virus neutralization data allowed us to sift through the myriad of virus quasi-species that evolved in this individual to identify envelope sequences from the nodes that appeared to define the transition from neutralization sensitive *envs* to the neutralization resistant *envs* that occur in EN plasma. Synthetic genes from these nodes were functional in infectivity assays and sensitive to neutralization by bNAbs, and may provide a novel source of immunogens for HIV vaccine development.

## Introduction

A major goal of HIV-1 vaccine research is to develop HIV envelope glycoprotein immunogens that will induce broadly neutralizing antibodies (bNAbs). The highest levels of bNAbs are found in the sera of rare individuals termed elite neutralizers (ENs). ENs are defined as HIV-1 infected individuals who possess antibodies capable of neutralizing tier 2 and 3 viruses from at least four different clades of virus at serum dilution titers of 1:300 or more [1]. Despite more than 30 years of research, none of the HIV vaccine antigens described to date are able to elicit antibody responses similar to those found in ENs. Moreover, no immunogen has been described that is able to consistently stimulate antibodies to more than one of the five main epitopes recognized by bNAbs thought to be required for protection [2–9]. Historically, a major hypothesis put forward to account for the inability of candidate HIV vaccines to elicit bNAbs was that the immunogens lacked the quaternary epitopes present only on the trimeric structures associated with spikes on the virus surface [10, 11]. Several groups have now developed trimeric forms of the HIV envelope protein that appear to closely resemble the spike proteins [12–16]. These proteins possess the epitopes recognized by all of the major classes of bNAbs, and thus represent a significant advance over the monomeric envelopes tested previously [17, 18]. However, several studies have reported that these trimers are unable to elicit bNAbs when used in immunization studies [14, 19–24]. Thus the epitopes recognized by bNAbs, even in trimeric structures, are poorly immunogenic and the current challenge for HIV vaccine development is to enhance the immunogenicity of epitopes recognized by bNAbs. The outcomes observed so far with the current trimeric vaccine immunogens may parallel the outcomes seen for most individuals infected with HIV whereby only 10–30% of infected individuals produce bNAbs [1, 25–28] despite being continuously exposed to properly-folded trimeric HIV envelope proteins. It is thus likely that only rare envelope proteins possess the specific biophysical features that define the immunogenic structure required to elicit the formation of bNAbs.

The goal of this study was to identify and recover the rare Env that possess the immunogenic structures able to stimulate the formation of bNAbs. By definition, these Envs were present in ENs and preceded the appearance of bNAbs in EN plasma. However, it is likely that these proteins are metastable and only transiently present in plasma. This conclusion is supported by the observations that bNAbs are only detected 2–3 years post infection [29–32] and that Envs from EN plasma are resistant to neutralization by autologous antibodies [29, 33]. Thus, identification of the *envs* with the optimal features to promote bNAb development is problematic and the challenge for immunogen selection is to sift through the multitude of sequences that evolve in each HIV infected individual to identify those that occur in the interval between the time when bNAbs first appear and the time when neutralization resistant viruses are detected in the plasma. Multiple structural features have been documented that can account for resistance to neutralization. These include point mutations at antibody contact sites, insertions and deletions resulting in conformational masking, and the insertion, deletion, or shifting of N-linked glycosylation sites (NGS) to destroy or shield epitopes recognized by glycan dependent antibodies [33–40].

One approach to identifying the transitional *env* sequences that elicited bNAbs would be to isolate those viruses from longitudinal samples obtained from an EN [30, 34, 41–47]. However longitudinal specimens are often not available, and the frequency between sampling of sequential specimens is often too long to capture the emergence of a transitional virus isolate. However, it might be possible to identify these interim sequences by computational methods. Since provirus DNA appears to immortalize the history of virus evolution within an individual [48–50], it should preserve the neutralization sensitive ancestral *env* sequences from ENs that promoted bNAb development before their elimination by immune selection. We reasoned that a phylogenetic analysis of plasma and proviral sequences combined with neutralization data from autologous plasma would allow us to infer the sequences of neutralization sensitive ancestral sequences that gave rise to bNAbs but have been eliminated from plasma by immune selection. Our results demonstrate that sequences from blood taken at a single time point could be used to generate phylogenetic trees that traced the sequential evolution of sensitivity and resistance to neutralization by bNAbs. Ancestral sequences occurring at nodes immediately prior to the appearance of neutralization resistant virus sequences could be identified by computational methods and used to create synthetic genes. These inferred sequences were functional in virus infectivity assays, retained neutralization sensitivity to neutralization by autologous antibodies, possessed multiple epitopes recognized by multiple prototypic bN-mAbs, and possessed unique structural features in regions of the Env protein known to possess epitopes recognized by bNAbs. The availability of these sequences will allow us to test the hypothesis that envelope proteins from ENs may possess unique immunogenic features that facilitate the formation of bNAbs.

## Materials and methods

### Collection of plasma and peripheral blood mononuclear cells (PBMCs) from individuals possessing bNAbs

Archival plasma samples (blinded) were provided by Global Solutions for Infectious Diseases (South San Francisco, CA). These were collected by a physician from volunteers attending a regional center for recruitment in the San Francisco Bay area. Prior to any screening procedures, each participant provided written informed consent, in accordance with a protocol for this study approved by Western Institutional Review Board (WIRB) (Puyallup, WA). Inclusion criteria stipulated HIV-positive men and women 18–65 years of age, HIV ELISA or Western Blot positive for at least one year prior to screening, and who have never received anti-retroviral therapy (including post-exposure prophylaxis). An initial 10 mL of blood was collected into two EDTA tubes and plasma were tested for bNAbs (described below). Four individuals with high titers of bNAbs were asked to participate in a 500 mL blood draw in a clinic setting. The blood was processed by a commercial laboratory using standard techniques and PBMCs and plasma were separated, aliquoted, and cryopreserved under conditions that preserved plasma virus RNA and cell associated provirus DNA. The specimens were aliquoted and stored at -80°C until further analysis.

### Virus neutralization assays and screening for bNAbs

The plasma were screened for virus neutralizing activity using the Monogram Biosciences HIV Neutralizing Antibody Assay (Monogram Biosciences, South San Francisco, CA). This assay is based on the pseudotype virus assays system described previously [29, 51] and has been used in multiple research studies [1, 52, 3], clinical studies, and studies used to detect bNAbs [53,54]. Initially, plasma were screened using a panel of five viruses that are highly predictive of the EN phenotype [1]. The screening assay included controls to detect both HIV-specific antibody mediated neutralization as well as non-specific neutralization resulting from serum factors or unreported treatment with anti-retroviral drugs. The plasma that exhibited neutralizing activity to the initial panel were further tested against a panel of 19 international isolates widely used in HIV vaccine research. For serum or plasma, the neutralizing antibody titer (ID_50_) is defined as the reciprocal of the plasma dilution that produces a 50% inhibition in target cell infection. For monoclonal antibodies or entry inhibitors, the neutralization titer (IC50) is defined as the reciprocal of the concentration (ug/mL) that produced a 50% inhibition of target cell infection. Values were determined from titration curves as described previously [29]. Viruses with mutations designed to confer resistance to neutralization by specific by bN-mAbs were tested for sensitivity to neutralization by EN1 serum by a standard TZM-bl neutralization assay [29].

### Recovery of plasma virus and provirus sequences

Full-length functional clones of HIV envelope genes were recovered from plasma virus RNA using the PhenoSense Entry assay system of Monogram Biosciences. Full-length functional clones of HIV envelope genes were recovered from provirus DNA in PBMCs using the Trofile DNA assay system of Monogram Biosciences. Both assays include a selective step to eliminate defective and non-infectious envelope sequences common in clinical specimens. Envelope genes recovered from plasma virus and provirus sequences were expressed as pseudoviruses and tested for sensitivity and resistance to neutralization by autologous polyclonal serum as well as a panel of broadly neutralizing antibodies provided by the NIH AIDS Reagent Program, Polymun Scientific (Vienna, Austria), and Dr. Dennis Burton (Scripps Clinic and Research Institute, La Jolla, CA). The neutralizing antibody titer (IC_50_) for monoclonal antibodies is defined as the concentration of purified mAb (μg/L) that produces a 50% reduction in target cell infection.

### Phylogenetic analyses

The plasma virus and provirus gp160 sequences were aligned in Geneious v 5.6.7 [55] using the Muscle algorithm and then edited manually. Insertions and deletions were removed from the alignment and Maximum Likelihood (ML) and Minimum Evolution (ME) gene trees were constructed using MEGA5 [56]. The ML tree was constructed using the Tamura 3-parameter model, with gamma distributed rate heterogeneity (six discrete categories) and a proportion of invariant sites (G+I). The sequence of the JRCSF Isolate of HIV was designated as the outgroup. A Bayesian tree was also constructed using MrBayes 3.1.2 [57], specifying a GTR (General Time Reversible) model with G+I, 5,000,000 generations and a burn-in of 12,500. Ancestral sequences at select nodes were reconstructed using the same program after removal of two duplicate plasma sequences and two sequences with early stops. These nodes were designated Elite Neutralizer 1-Ancestral Node 1 (EN1-AN1) and Elite Neutralizer 1-Ancestral Node 2 (EN1-AN2). The co-receptor tropism of native and inferred envelope sequences was determined with the Trofile or Trofile DNA assay tests (Monogram Biosciences). In this assay envelope sequences are classified as CCR5- tropic, CXCR4 tropic, or dual or mixed tropic (DM) as described previously [58, 59].

### Statistical analysis of physical properties

Lengths of variable (V) and constant (C) regions and the number of predicted N-linked glycosylation sites (PNGS) were determined by manual inspection. Statistically significant differences in V and C region lengths and the number of glycosylation sites statistics were compiled using the non- parametric Mann-Whitney U test (GraphPad PRISM 6, GraphPad Software Inc., La Jolla, CA).

### Expression of recombinant gp120

Codon optimized genes were synthesized (Invitrogen Inc., Waltham, MA) for *envs* selected for protein production. The synthetic genes were cloned and expressed in 293 HEK cells (FreeStyle 293F; Invitrogen, Inc., Waltham, MA) or in 293HEK cells deficient in the production of N-acetylglucosaminyltransferase-1 (GnT1^-^) cells [60] as chimeric transcription units using a cytomegalovirus (CMV) promoter as described previously [60–63]. Transfections were performed with the MaxCyte electroporation system using the HEK293 protocol (MaxCyte STX, MaxCyte Inc., Gaithersburg, MD). All constructs were expressed as fusion proteins that possessed an N-terminal tag epitope of 27 amino acids from herpes simplex virus 1 glycoprotein D (gD-1) as described previously. The gD-1 tag epitope facilitated purification by immunoaffinity chromatography [63–65]. Proteins recovered from immunoaffinity chromatography were further purified by size exclusion chromatography (Sephacryl S-200, GE Healthcare Biosciences, Pittsburgh, PA) and the purity of the recovered proteins was assessed by scanning densitometry after sodium dodecyl sulfate polyacrylamide gel electrophoresis (SDS-PAGE) using NuPAGE 4–12% Bis-Tris precast gels (Thermo Fisher Scientific, Invitrogen, Carlsbad, CA) in MES running buffer (Thermo Fisher) and stained with SimplyBlue stain (Thermo Fisher).

### Antibody binding assays

The binding of antibodies to recombinant proteins was carried out by Fluorescence Immunoassay (FIA). Briefly, 2 ug/mL of anti-gD tag monoclonal antibody, 34.1, was diluted into PBS and incubated at room temperature overnight in 96 well black-microtiter plates (Greiner, Bio-One, USA). Plates were blocked in PBS containing 5% BSA and then incubated with either 1:5 or 1:2 diluted supernatants. Captured antigen was then incubated in 3 fold dilutions of primary antibody, followed by incubation with a 1:3,000 dilution of goat-anti-human AlexaFluor 488 conjugated polyclonal (Jackson ImmunoReagents). All incubations were carried out for 1.5 hours at room temperature, followed by a 4x wash in PBST buffer. Absorbance was read using an EnVision Multilabel Plate Reader (PerkinElmer, Inc Waltham, MA) with a FITC 485 excitation filter and a FITC 353 emission filter.

### Accession numbers

The EN1 sequences described in this publication have been deposited in Genbank with accession numbers for nucleotide and amino acid sequences as follows: MK164661-MK164683.

## Results

### Screening plasma from anti-retroviral treatment-naïve subjects to identify ENs

To identify ENs, we screened de-identified plasma from 20 anti-retroviral drug treatment-naïve individuals collected in 2009. The plasma were tested for virus neutralizing activity (Table 1) using a panel of five viruses that are highly predictive of the EN phenotype [1]. Plasma that exhibited neutralizing activity to the initial panel were further tested against a panel of 19 international isolates widely used in HIV vaccine research (Table 1). We found two individuals (001 and 015) who possessed high levels of bNAbs that met the definition of the EN phenotype, and several other individuals (007, 012, and 017) that exhibited considerable breadth of neutralization but did not meet the definition of an EN. Subject 001 exhibited the highest overall titers and was able to neutralize viruses from clades A, B, C, D, and CRF01_AE.

**Table 1:** Screening of sera from HIV-infected subjects to identify elite neutralizers.

		Neutralization titers (ID_50_^a^)														
No.	Sera/virus	Clade	1	4	5	6	7	8	10	11	12	14	15	17	N16	Z23
1	94UG103	A	**785**	32	36	**69**	**81**	**57**	**31**	70	**43**	61	**433**	**135**	30	121
2	92BR020	B	**1241**	**121**	**219**	**111**	**267**	**108**	**44**	140	**194**	67	**292**	**185**	**65**	**272**
3	93IN905	C	**986**	**78**	**116**	**124**	**153**	**85**	**141**	**361**	**658**	**122**	**239**	**287**	**91**	**326**
4	M-C-026	C	**507**	55	**66**	**91**	**219**	**72**	**38**	113	**300**	**331**	**316**	**112**	**55**	**277**
5	92TH021	AE	**408**	53	44	**109**	**95**	**55**	**44**	41	**449**	**103**	**1,406**	**138**	**57**	**255**
6	SF162	B	**5611**	**9369**	**76894**	**5529**	**51234**	**1620**	**2487**	**1429**	**9483**	**2780**	**18938**	**14287**	**4720**	**22535**
7	1196	B	**3404**	**126**	**161**	**141**	**265**	**189**	**59**	**169**	**105**	**86**	**626**	**927**	**142**	**413**
8	TRO	B	**2556**	46	**49**	**89**	**167**	**89**	**54**	89	**119**	61	**369**	**129**	**63**	**297**
9	JRFL	B	**4761**	31	40	**56**	**122**	**974**	32	47	**47**	**876**	**335**	**180**	34	**514**
10	BG1168	B	**224**	24	38	37	**47**	37	<20	35	**30**	**69**	**170**	**136**	21	120
11	QHO692	B	**720**	26	27	**60**	34	39	30	42	**35**	36	**147**	**90**	24	137
12	REJO	B	**1206**	**66**	**59**	**126**	**369**	**148**	39	34	**88**	**302**	**1,029**	**196**	**62**	**515**
13	M-SC-B-006	B	**382**	21	<20	34	39	**81**	21	32	**59**	45	**128**	**109**	<20	<100
14	APV-16	B	**1101**	36	46	**66**	**100**	**61**	**56**	87	**112**	39	**80**	**223**	**90**	**572**
15	M-Chr-B-013	B	**381**	42	34	**57**	**92**	**51**	43	49	**49**	40	**639**	**75**	23	189
16	PVO	B	**2328**	37	**72**	**65**	**84**	**77**	**64**	50	**95**	**78**	**332**	**125**	28	**439**
17	M-A-002	A	**291**	29	24	32	**72**	**55**	**28**	59	**42**	46	35	83	22	181
18	M-A-006	A	**952**	24	22	38	**54**	40	**42**	60	**131**	**82**	**105**	**104**	<20	**230**
19	M-C-003	C	40	25	<20	31	**32**	28	22	47	**98**	41	46	**111**	23	<100
20	M-C-020	C	**1075**	32	<20	43	**72**	39	**31**	66	**111**	71	**228**	**99**	<20	<100
21	M-D-006	D	**2504**	28	35	50	**60**	43	<20	38	20	37	**314**	78	<20	**238**
22	M-D-009	D	**1465**	**278**	**126**	**222**	**712**	**382**	**140**	124	**83**	**144**	**905**	**209**	**135**	**476**
23	JRCSF	B	**5933**	54	**59**	**100**	**139**	**116**	<20	46	**95**	**193**	**788**	**178**	**177**	**402**
24	JRCSF	B	**4995**	43	**54**	**84**	**137**	**133**	<20	69	**120**	**293**	**1,045**	**201**	**172**	**385**
25	NL43	B	**2131**	**433**	**271**	**844**	**1394**	**464**	**97**	**232**	**1,852**	**486**	**1,182**	**1,361**	**769**	**3598**
26	NL43	B	**1947**	**392**	**203**	**888**	**1390**	**931**	**93**	**345**	**2,448**	**847**	**1,733**	**2,217**	**591**	**3172**
27	aMLV	—	<20	<20	<20	<20	<20	<20	<20	64	<20	25	23	28	<20	<100

^a^The neutralizing antibody titer (ID_50_) is defined as the reciprocal of the plasma dilution that produces a 50% inhibition in target cell infection. Values in bold represent neutralization titers that are at least three times greater than those observed against the negative control (aMLV). All clones tested were CCR5 tropic. All human sera/plasma were treated to minimize non-specific backgrounds. Viruses 1–5 are from the Simek panel which defines the EN phenotype [1].

Based on these results, we requested an additional sample of blood for plasma and PBMC isolation from this subject, designated EN1. To further explore the potency of the neutralizing activity in EN1, the plasma was screened for neutralizing activity against a panel of viruses that included isolates or mutants known to be resistant to neutralization by several prototypic bN-mAbs, including PG9, PG16, and VRC01. Remarkably, EN1 serum was effective in neutralizing nine of ten viruses resistant to neutralization by the VRC01 bN-mAb (Table 2) which targets the CD4 binding site [66]. This bN-mAb is currently being tested as a therapeutic agent to suppress and/or clear HIV infections [67]. The plasma was also effective in neutralizing 8 of 9 viruses resistant to neutralization by PG9, a bN-mAb, that requires mannose-5 at positions N156 and N160 in the V1/V2 domain for binding, and 6 of 7 viruses resistant to neutralization by PG16, a bN-mAb that overlaps the PG9 epitope and requires both simple and complex glycans for binding [68]. These results demonstrate that the EN1 serum possessed unusually broad and potent virus neutralizing activity.

**Table 2:** Serum antibodies from EN1 are effective against neutralization-resistant viruses.

No.	Virus/mutant	Clade	Serum neutralization titer (ID_50_)	bN-mAb neutralization (IC_50_)		
			EN1	PG9	PG16	VRC01
1	Du422.1	C	**54**	**0.60**	**0.10**	>50
2	CAP210.2.00.E8	C	**151**	**0.30**	**<0.02**	>50
3	TV1.21	C	**222**	**<0.02**	**<0.02**	>50
4	T278.50	CRF02_AG	**553**	**0.50**	**0.30**	>50
5	CAP45.2.00.G3	C	**231**	**<0.02**	**<0.02**	**28.30**
6	CAP45.2.00.G3/N160A		**104**	>50	>50	**28.20**
7	TRO.11	B	**1571**	>50	**10.00**	**0.30**
8	TRO.11/N322A		**421**	>50	**5.27**	**0.75**
9	AC10.0.29	B	**240**	**0.10**	**<0.02**	**1.30**
10	AC10.0.29/N332A		**284**	**0.20**	**<0.02**	**1.90**
11	172284_017	B	**1012**	>10	>10	>10
12	172284_036		**289**	>10	>10	>10
13	108051_005	B	<100	>10	>10	>10
14	108051_005_D180N		**441**	>10	>10	>10
15	172950_007	B	**671**	**4.25**	**2.51**	>10
16	172950_007N197H		**4556**	>10	>10	>10
17	MN-3	B	**1850**	>20	>20	**>0.02**
18	JRCSF	B	**1597**	**0.01**	**0.01**	**0.36**
19	aMLV-SVA	Control	<20	>50	>50	>50

Viruses/mutants represent pseudoviruses prepared using cloned envelope glycoprotein genes. Pairs of viruses from CAP45, TRO.11, AC10, 108051 and 172950_007 differ by single amino acid mutations that confer sensitivity and resistance to various bN-mAbs. Viruses 172284_017 and 172284_036 were neutralization resistant clinical isolates. For serum, virus neutralization represents the reciprocal of the serum dilution that results in 50% inhibition (ID_50_) of target cell infection. For mAbs, virus neutralization represents the concentration of bN-mAbs (μg/mL) that results in 50% inhibition (IC_50_) of target cell infection. Values in bold represent significant results where neutralization titers that are at least three times greater than those observed against the negative control (aMLV). MN-3 and JRCSF were included as neutralization sensitive positive controls.

### Sequencing and phylogenetic analyses

**Fig 1:**
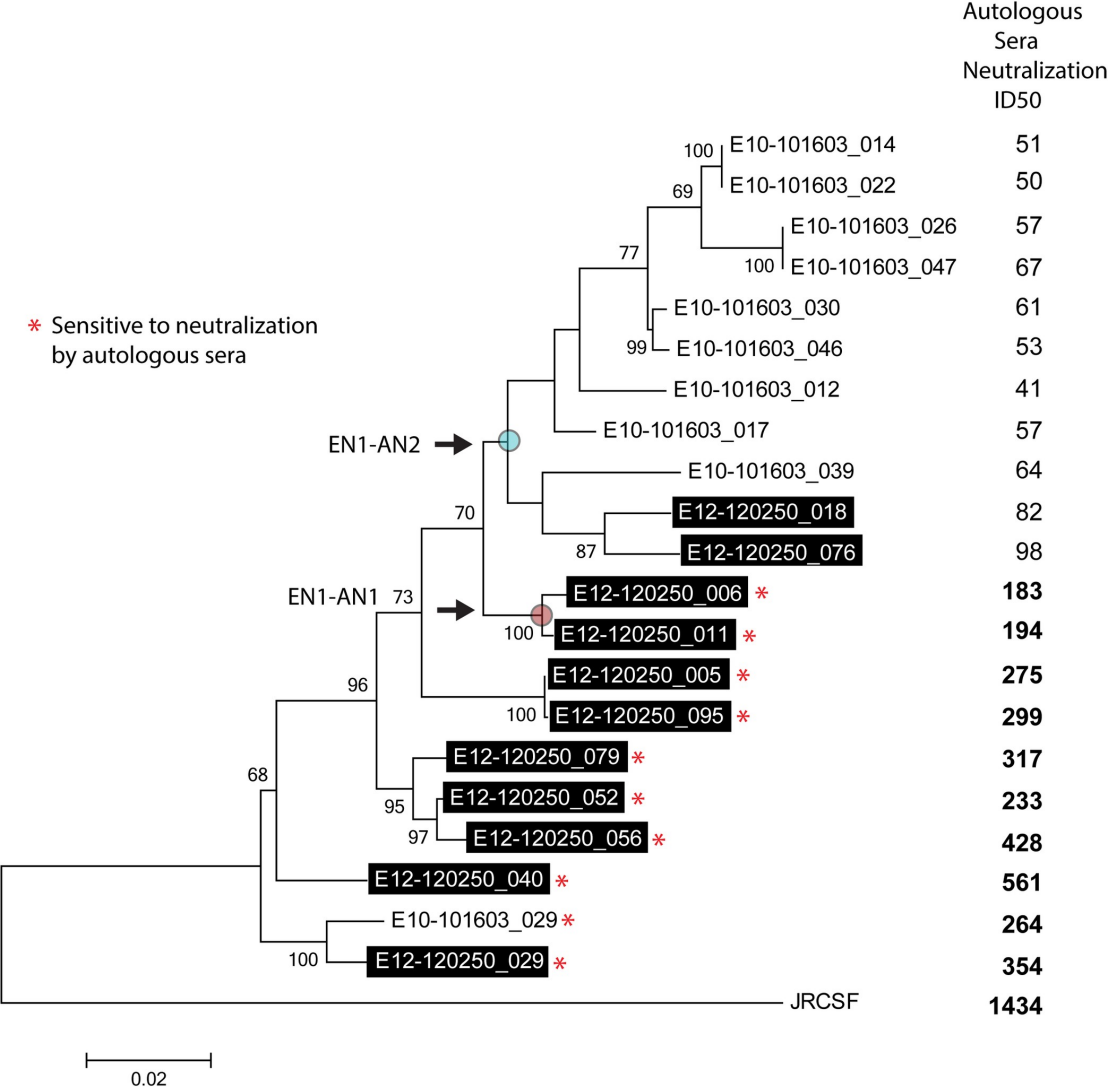
Maximum likelihood (ML) phylogenetic tree tracing the evolution of envelope glycoprotein sequences in EN1. Data are for provirus gp120 sequences (boxed, shaded labels) and plasma virus sequences showing bootstrap support values exceeding 60% (based on 1000 replicates). Asterisks (*) indicate envelopes sensitive to neutralization by autologous sera. Also shown are neutralization titers (ID_50_) for each envelope sequence using autologous (EN1) serum. Values in bold represent significant neutralization. The positions of the **r**econstructed ancestral nodes (EN1-AN1 and EN1-AN2) are indicated as labeled.

In order to define the *env* sequences that evolved in EN1, we recovered ten full-length functional gp160 genes from plasma virus RNA and eleven genes from cryopreserved PBMCs collected at the same time point. A caveat of obtaining sequences from provirus DNA is that they are often defective and have mutations that result in non-infectious viruses. We circumvented this problem by using the Monogram Biosciences Trofile DNA assay system that includes an infectivity screen that eliminates defective provirus *env* sequences. Phylogenetic trees were constructed using three different approaches which yielded identical topologies with the exception of two sequences (E10-101603_ 030 and E10-101603_017) that clustered together in the ME tree but not in the ML or Bayesian trees. This minor change did not impact the interpretation of the data. The resulting ML tree is shown in Fig 1.

### Neutralization of plasma virus and provirus clones by autologous plasma

Virus neutralization studies were also carried out using autologous contemporaneous plasma [69] and the resulting neutralization titers were aligned with the position of each sequence on the phylogenetic tree (Fig 1). We found that the neutralization resistant sequences were primarily associated with the plasma virus derived sequences (E10-101603 clones) and formed a distinct clade with the exception of a single neutralization-sensitive clone (E10-101603_029) recovered from plasma that clustered with the provirus sequences (E12-120250 clones) at the base of the tree. The position of this clone, E10-101603_029, is strongly supported by bootstrap support values of 100 and Bayesian posterior probabilities of 1 (not shown).

Similarly most of the provirus sequences were sensitive to neutralization by autologous plasma with neutralization titers ranging from 1:183–1:561. We found that two provirus clones that were resistant to neutralization by autologous plasma (E12-120250_018 and E12-20250_076) clustered with the resistant plasma virus sequences. Despite these departures from strict monophyly for plasma and proviruses, neutralization sensitive and resistant viruses segregated into two distinct clades and the mean autologous serum neutralization titers of the combined sensitive viruses differed significantly from the resistant viruses (Mann Whitney U test, *P* < 0.0001****).

### Neutralization of plasma virus and provirus clones by bN-mAbs

**Fig 2:**
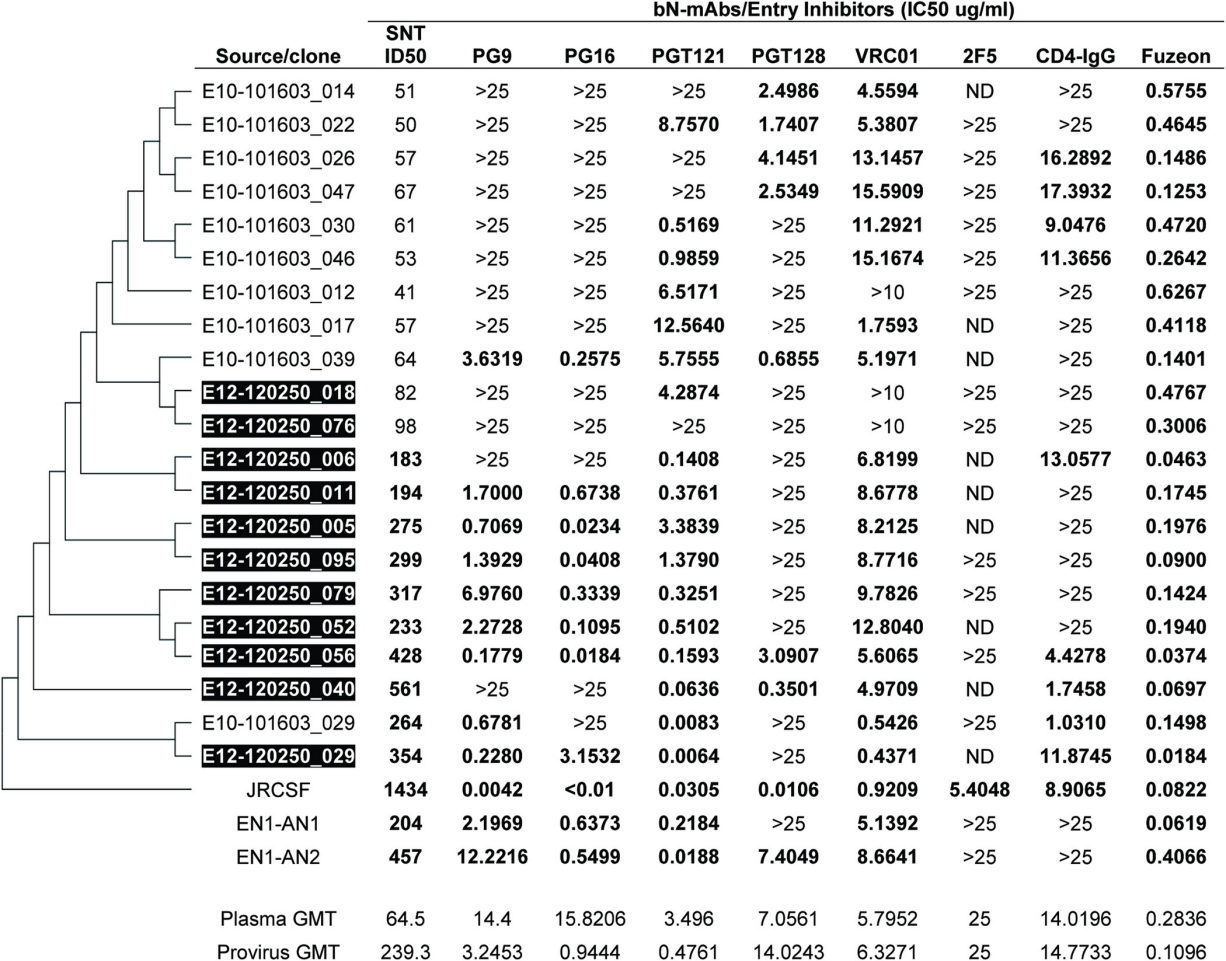
Sensitivity of EN1 provirus and plasma virus clones to neutralization by autologous serum and bN-mAbs presented in the order of the phylogenetic tree. Source/clone represents pseudoviruses prepared using cloned envelope glycoprotein genes derived from plasma virus RNA or provirus DNA (highlighted in black). For serum, neutralization titers (SNT) indicate the reciprocal of the serum dilution that results in 50% inhibition (ID50) of target cell infection. For mAbs, neutralization titers indicate the concentration (μg/mL) of bN-mAbs (IC50) that results in 50% inhibition of target cell infection. Values in bold represent significant neutralization titers that are at least three times greater than those observed against the negative control (aMLV). ND indicates assay not done. Values >10 and >25 indicate no neutralization at concentrations of bNAbs of 10μg/ml and 25 μg/ml, respectively.

**Fig 3:**
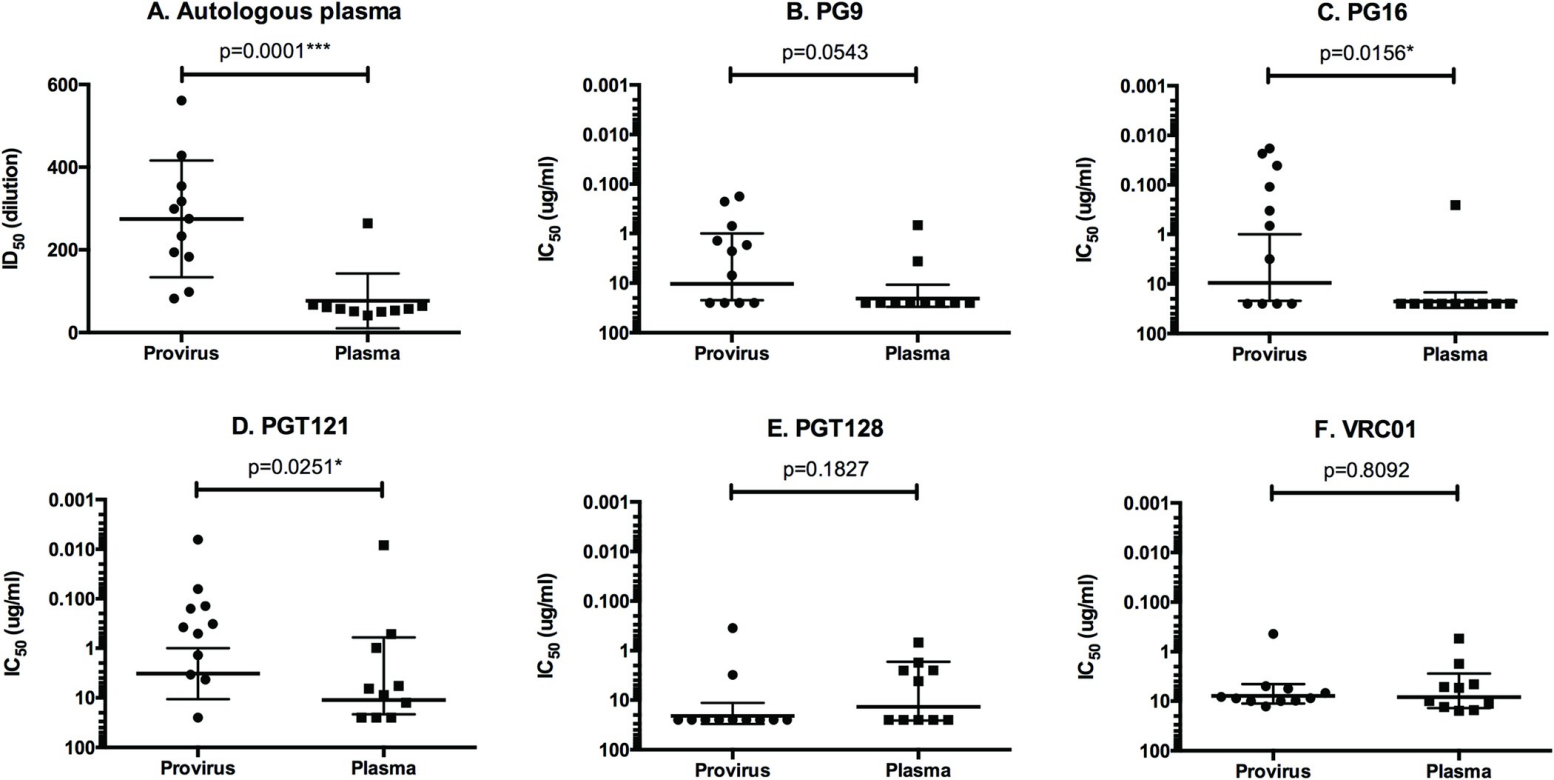
Differences in the sensitivity to neutralization by autologous plasma and bN-mAbs between plasma and provirus derived pseudovirions from EN1. The virus neutralization data from Fig 2 were plotted as a function of the source of virus (plasma virus RNA or provirus DNA), and the geometric means of titers were calculated. The distributions were compared by unpaired, nonparametric Mann Whitney tests. *P* values for differences in neutralization titers are indicated.

Plasma virus sequences were also analyzed for sensitivity and resistance to neutralization against a panel of prototypic bN-mAbs (Figs 2 and 3). We found that eight of ten plasma clones were resistant to neutralization by PG9 and nine of ten clones were resistant to neutralization by PG16. While this difference in neutralization sensitivity was significant for PG16 (P = 0.0156), there was a trend to significance for PG9 (P = 0.0543). Both antibodies are known to bind to overlapping glycan dependent epitopes in the V1/V2 domain [52, 70]. We found that both plasma viruses and proviruses were sensitive to neutralization by the PGT121 bN-mAb that recognizes a glycan dependent epitope at the base of the V3 domain. However the provirus derived clones were significantly more sensitive to neutralization than the plasma virus derived sequences (P = 0.0251). Although the epitope recognized by the PGT128 bN-mAb overlaps with PGT121, the pattern of neutralization we observed with these two antibodies differed considerably. Thus no significant differences were observed between plasma virus and provirus clones with respect to neutralization sensitivity by PGT128 (P = 0.1827). Although viruses sensitive to neutralization by the VRC01 bN-mAb were found among clones from both plasma viruses and proviruses, the neutralization titers were comparatively low and not significantly different (P = 0.8092). It is known that the VRC01 bNAb overlaps the CD4 binding site and it was interesting to note that both the plasma viruses and the proviruses were similarly resistant to neutralization by the CD4-IgG entry inhibitor [71,72]. In contrast, all of the clones were sensitive to neutralization by the Enfuvirtide entry inhibitor (Fuzeon, Roche, South San Francisco, CA) that targets sequence in gp41. All of the viruses tested were resistant to 2F5, a bN-mAb that targets the membrane proximal external region (MPER) in gp41. Overall, this study confirmed previous reports [29, 73, 74] that circulating plasma viruses from ENs are largely resistant to neutralization by autologous serum. However, we found that the plasma viruses from EN1 differed considerably in their sensitivity to neutralization by bN-mAbs. The single plasma virus clone with sensitivity to EN1 plasma (E10-101603_029) was also highly sensitive to PGT121 and moderately sensitive to PG9, VRC01, and CD4-IgG.

### Identification and recovery of sequences at ancestral nodes for infectivity and neutralization studies

Our phylogenetic analyses (Fig 1) identified a series of ancestral nodes that gave rise to the specific *envs* recovered. Two of these nodes (EN1-AN1 and EN1-AN2) represent common ancestors of virus sequence present before and after the evolution of resistance to neutralization by autologous plasma. The inferred sequence at the EN1-AN1 node represents a progenitor of the two final neutralization sensitive provirus sequences. The EN1-AN2 node was a progenitor of the neutralization resistant clade which was comprised of a mixture of plasma and provirus. The position of these two nodes are at a transition between sampled sequences which differ by either loss or masking of known bN-mAb epitopes as indicated by the independent neutralization by PGT121, PG9 and PG16 (Fig 2). According to our hypothesis, the sequences at these nodes should more closely resemble the immunogenic variants that stimulated the formation of bNAbs in autologous serum that resulted in the selection of viruses resistant to neutralization by autologous plasma.

**Fig 4:**
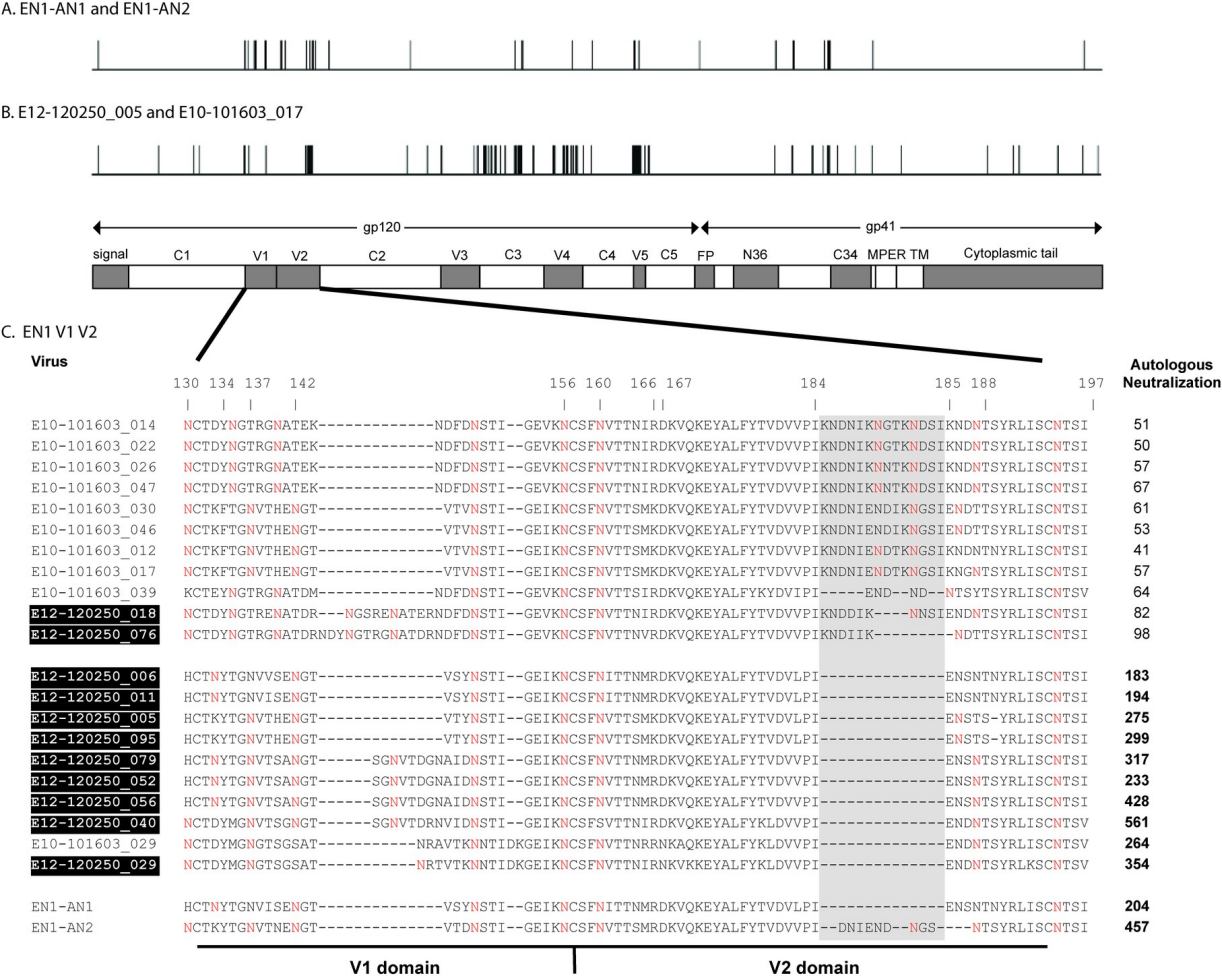
Characterization of the locations of sequence differences between inferred ancestral envelope proteins and authentic envelope proteins from EN1. The location and number of sequence differences between the inferred ancestral EN1-AN1 and EN1-AN2 envelope proteins (A) and the authentic envelope proteins (E12-120250_005 and E10-101603-017) (B) are represented in horizontal diagrams of gp160 structure. EN1-AN1 and EN1-AN2 represent common ancestors of virus sequence present before and after the evolution of resistance to neutralization by autologous plasma. The E12-120250_005 and E10-101603_017 sequences are representative of authentic sequences from the most recently evolved clades of viruses sensitive and resistant to neutralization by contemporaneous autologous EN1 serum. (C) Alignment of V1/V2 sequences from EN1 highlighting V2 insertion in relation to autologous neutralization titers. Neutralization resistance in EN1 correlated with a 9–14 amino acid insertion containing two N-linked glycosylation sites between positions 184 and 185 in the V2 domain (gray shading). Shaded boxes indicate provirus sequences, unshaded labels indicate plasma virus sequences. Values in bold represent significant neutralization, as described in Table 1. EN1-AN1 and EN2-AN2 represent the inferred sequences at nodes indicated in Fig 1. Red Ns (N), indicate the locations of predicted N-linked glycosylation sites. Amino acid positions are designated with reference to the prototypic HXB2 envelope sequence.

Comparison of the inferred EN1-AN1 and EN1-AN2 sequences showed that they differed by 29 amino acids (Kimura 2-parameter distance = 0.0126) with 13 of the mutations occurring in the V1/V2 region (Fig 4A). Three of these differences were related to rearrangements of the glycan profile in the V1 domain at positions 130, 133 and 137. In addition we noted a nine amino acid insertion in EN1-AN2 between amino acids 184 and 185 in the V2 domain that appeared to be an intermediate step in the creation of the 14 amino acid insertion that was associated with neutralization resistance and added one additional PNGS site. V3 sequences between the two reconstructions were identical. For comparison, the location of polymorphisms between two clones that flanked the inferred ancestral sequences in the phylogenetic tree (e.g., the neutralization sensitive E12-120250_005 and the neutralization resistant E10-101603_017) are provided in Fig 4B. These differed by 68 mutations (Kimura 2-parameter distance = 0.0397) with six differences occurring between the V1/V2, three in the V3, 16 in the C3, and ten in the V4. The E10-101603_017 clone possessed a V2 insertion of 15 amino acids, a two residue deletion in the V4, and a nine residue deletion in the V5. The ancestral reconstruction method thus reduced the number of sequence differences observed at the transition point between the neutralization sensitive and resistant clades compared to simply contrasting sequences recovered from authentic viruses. The concentration of mutational differences between the two reconstructions suggested that the V1/V2 may harbor key immunogenic features related to the transition from autologous sensitivity to resistance.

### Expression, tropism, and neutralization sensitivity of ancestral sequences

The ancestral EN1-AN1 and EN1-AN2 sequences (Fig 1) were chemically synthesized and expressed as pseudoviruses. We found that both genes encoded envelopes that were functional and preserved resistance to neutralization by high concentrations of CD4-IgG (>25 μg/ml) (Fig 2). Resistance to this concentration of CD4 is typical of clinical isolates of HIV-1 and differs markedly from lab adapted, tier 1 viruses [72, 75]. We found that the EN1-AN1 virus was somewhat dual tropic, exhibiting weak infectivity in CXCR4 cells, but strong infectivity in CCR5 expressing cells (Table 3). In contrast, the EN1-AN2 virus was exclusively CCR5 tropic and showed no infectivity in CXCR4 positive cells. In a control experiment, we also examined the tropism of one of the most neutralization resistant viruses in the panel of plasma virus sequences (E10-101603_17) and found that it also exclusively depended on CCR5 for infectivity.

**Table 3:** Infectivity of inferred ancestral neutralization-sensitive and resistant *env*s, EN1-AN1 and EN1-AN2, compared to the representative plasma virus clone E10-101603_017.

Virus Name	Autologous serum neutralization	Tropism	RLU	
			R5	X4
EN1-AN1	1:204	DM	4	1
EN1-AN2	1:457	R5	3	0
E10-101603_017	1:57	R5	3	0

For serum neutralization, data indicate the reciprocal of the serum dilution that results in 50% inhibition (ID_50_). For receptor tropism assays, the infectivity of pseudoviruses expressing luciferase on cells expressing CD4 and the chemokine receptors CCR5 (R5) or CXCR4 (X4)in relative luminescence units (RLUs) were measured according to the following scale: 0: < 5,000 RLU; 1: 5,001 to 15,000 RLU; 2: 15,001 to 150,000 RLU; 3: 150,001 to 1,000,000 RLU; 4: >1,000,000 RLU. DM indicates dual/mixed tropism.

When we examined the sensitivity of EN1-AN1 and EN1-AN2 to autologous serum and to a panel of bN-mAbs, both displayed the neutralization sensitive phenotype typical of provirus sequences (Fig 2). We found that both were sensitive to neutralization by PG9, PG16, PGT121, VRC01, and Fuzeon (Enfuvirtide). However, the EN1-AN1 virus was resistant to neutralization by PGT128 whereas the EN1-AN2 virus was sensitive to neutralization by this antibody. Additionally the EN1-AN2 virus was 10 fold more sensitive to neutralization by PGT121 than EN1-AN1. Overall, the EN1-AN2 sequence appears to have features that confer bN-mAb sensitivities that are intermediate to the majority of plasma virus and provirus sequences, and retains the epitopes for sensitivity to bNAbs in autologous serum.

### Physical properties of sequences recovered from EN1

An analysis was carried out to characterize the physical properties of the EN1 Envs that differed in their sensitivity to neutralization by antibodies in autologous serum (Table 4). This analysis included quantitation of the lengths and number of glycosylation sites in the five variable (V) and four of the conserved (C) domains of gp120 [76]. We found statistically significant differences between the lengths of gp120s from neutralization sensitive and resistant gp120 *envs* (P = 0.0324), but there was no significant difference between their glycan content. Further analysis showed that the differences in lengths could be attributed to differences in the number of amino acids in the V1 domain (P<0.0001). Significant differences in the number of glycosylation sites occurred in the V2, V4, and C4 domains. The combined V1/V2 domains of the neutralization sensitive proviruses were found to be, on average, ten amino acids shorter, with two less PNGS than the neutralization resistant plasma viruses. On a domain-by-domain basis, the lengths of the V2 domains of the neutralization sensitive viruses were approximately 12 amino acids shorter and had 1.4 fewer PNGS than the neutralization resistant viruses, but no significant differences were detected between the lengths and number of PNGS of the V1 domains. However, we noted considerable variation in the location of glycosylation sites between amino acids 130 and 140. PNGS in this region have been identified as contact residues for bN-mAbs, particularly N137 that is associated with PGT121 neutralization and binding [77, 78]. Significant differences were also observed between sensitive and resistant viruses for the V4 and C4 domains with an average 1.7 more PNGS in the V4 domain of neutralization-sensitive viruses compared to the neutralization-resistant viruses. The V4 has been shown to be an early target of autologous Nabs [79] and has glycan contacts for the PGT135 bN-mAb (N386 and N392) [80]. In the C4 domain the neutralization sensitive envelopes had, on average, 0.5 fewer PNGS than the resistant viruses (Table 4).

**Table 4:** Comparison of the physical characteristics of gp120 from the neutralization-sensitive and resistant clones from EN1.

Domain	Sensitive (S) Resistant (R)	Domain Length (Amino Acid) Mean ±SD	Range	*P* Value	Glycan Count Mean ±SD	Range	*P* Value
gp120	S	517.5±5.3	511–524	**0.0324**	25.6±3.1	21–29	0.4236
	R	524.3±5.6	519–533		27.3±1.7	24–30	
C1	S	100±0.0	100–100	>0.9999	1.7±0.5	1–2	0.2105
	R	100±0.0	100–100		2.0±0.0	2–2	
V1/ V2	S	70.0±4.1	65–74	**<0.0001**	6.6±1.2	5–8	**0.0030**
	R	80.7±4.1	72–87		8.4±1.1	6–10	
V1	S	31.2±3.8	27–35	0.7311	4.6±1.1	3–6	0.8142
	R	30.1±5.4	27–41		4.4±0.9	4–6	
V2	S	38.8±0.4	38–39	**<0.0001**	1.7±0.5	1–2	**0.0004**
	R	50.6±3.9	44–53		3.1±0.8	2–4	
C2	S	99±0.0	99–99	>0.9999	5.3±0.5	5–6	>0.9999
	R	99±0.0	99–99		5.3±0.5	5–6	
V3	S	35.0±0.0	35–35	>0.9999	0.8±0.4	0–1	0.4737
	R	35.0±0.0	35–35		1.0±0.0	1–1	
C3	S	52.0±0.0	52–52	<0.9999	3.2±0.4	3–4	0.4999
	R	52.0±0.0	52–52		3.3±0.7	2–4	
V4	S	33.8±1.7	31–35	0.0772	5.1±1.0	4–6	**0.0011**
	R	31.7±3.2	28–35		3.4±0.7	3–5	
C4	S	41.0±0.0	41–41	>0.9999	0.9±0.3	0–1	**0.0217**
	R	41.0±0.0	41–41		1.4±0.5	1–2	
V5	S	15.7±3.6	42664	0.3735	2.0±1.2	1–4	0.3029
	R	14.3±2.6	42725		2.3±0.7	2–4	

Data was collected from ten neutralization sensitive (S) and 11 neutralization-resistant (R) viruses shown in Table 3. The lengths and number of glycosylation sites in variable (V) and conserved (C) domains is indicated. Statistically significant differences in domain lengths and numbers of glycosylation sites were determined using the Mann-Whitney U-test with *P* values indicated. Statistically significant differences are indicated by *P* values in bold.

### Features that correlate with neutralization sensitivity and resistance

We next carried out an analysis of features that correlated with sensitivity and resistance to neutralization using the same method shown in Fig 3. An alignment of the V1/V2 sequences of the provirus and plasma virus clones (Fig 4C) identified a 9–14 amino acid insertion in the V2 domain between positions 184 and 185 that is highly correlated with resistance to neutralization by antibodies in autologous plasma. Further analysis showed that this insertion was highly correlated with resistance to the PG9, PG16, and PGT121 bN-mAbs (Table 5, S1A Fig). This insertion is accompanied by the incorporation of either one or two PNGS. This segment of the V2 connects the C and D strands of the four-stranded V1/V2 domain β-sheet structure and is disordered in the crystal structures of 3U2S [70], 3U4E [70], and 4NCO [81]. Although polymorphisms in the vicinity of positions 184–185 are not known to possess contact sites recognized by the PG9, PG16, or PGT121 bN-mAbs, several polymorphisms at position 184 have been reported to modestly affect neutralization sensitivity [37].

**Table 5:** Summary of features which have a significant association with sensitivity or resistance to autologous plasma or bN-mAb neutralization of EN1 viruses.

Feature in authentic sequences	Response to autologous plasma	EN1 plasma	Feature present in inferred sequences		Response to bN-mAb	bN-mAbs (P value)				
			AN1	AN2		PG9	PG16	PGT121	PGT128	VRC01
+V2 insertion	Resistant	**<0.0001**	-	+	Resistant	**0.0006**	**0.0043**	**0.0001**	0.3132	0.1512
+N444	Resistant	**0.0035**	-	-	Resistant	**0.0287**	0.0503	**0.0298**	0.1129	0.0944
+N402	Sensitive	**0.0017**	-	-	Sensitive	**0.0025**	**0.0104**	**0.0087**	0.4974	0.3094
+N130	Resistant	**0.0290**	-	+	Resistant	**0.0068**	**<0.0001**	0.1338	0.5622	0.9718
+N339	Sensitive	**0.0007**	-	-	Sensitive	**0.0375**	**0.0010**	0.1079	0.9447	>0.9999
+N337	Resistant	**0.0290**	+	+	Resistant	0.2284	**0.0196**	0.5572	0.6360	0.5945
+N137	None	0.0720	-	+	Sensitive	0.0690	0.1554	**0.0172**	0.1180	0.6011
+N295	Sensitive	**0.0392**	-	-	None	0.4160	0.1041	0.6617	0.4665	0.4215
+N413	Sensitive	**0.0353**	+	+	None	0.6057	0.1291	0.4308	0.1180	0.9169

PNGS features have HXB2 numbering. The associated response (sensitive, resistant or none) to each feature by autologous plasma or bN-mAbs was calculated by comparing the mean neutralization titers of viruses exhibiting the sequence feature to viruses that did not have the sequence feature (Mann-Whitney U-test (p = <0.05). Statistically significant responses are indicated by P values in bold.

We next used this approach to examine the effect of polymorphism at other PNGS on sensitivity and resistance to virus neutralization. We observed that the occurrence of a PNGS at position N444, like the insertion between positions 184 and 185, significantly correlated with neutralization inhibition by autologous plasma as well as the PG9 and PGT121 bN-mAbs (Table 5, S1B Fig). Thus this polymorphism may in some way, perhaps by steric hindrance, occlude the epitopes recognized by PG9 and PGT121 that occur in the V1/V2 and/or V3 stem regions.

Conversely, we found that the presence of a PNGS at position 402 in the V4 domain correlated with significantly improved neutralization by autologous plasma as well as the PG9, PG16, and PGT121 bN-mAbs, but not PGT128 or VRC01. This result (Table 5, S1C Fig) suggests that there may be a yet to be defined neutralizing antibody that depends on glycosylation at N402 for binding or that glycosylation at this position in some way affects the structure of gp120 to promote accessibility to the epitopes of PG9, PG16, and PGT121. This polymorphism does not seem to cause a major conformational change as has been observed previously [82] since it does not affect neutralization by VRC01.

Two other PNGS polymorphisms that correlated with resistance to neutralization by autologous plasma and one or more of the PG9, PG16, or PGT121 bN-mAbs (Table 5, S1D Fig, S1F Fig) included positions N130 and N337. Based on published structures, PNGS at these positions have the potential to occlude epitopes in the V1/V2 and V3 stem regions. Three polymorphisms correlated with sensitivity to neutralization included PNGS at positions 339 (S1E Fig), 295 (S1H Fig) and 413 (S1I Fig) which may be contacts for bNAbs that are promiscuous in their requirement for specific glycan structure [77, 83]. A PNGS at 413 has been associated with individuals that develop broadly neutralizing antibodies [84–86]. N137, a V1 glycan that was shown to be important for regulating affinity maturation of PGT121 [78], correlated significantly only with PGT121 neutralization (Table 5, S1G Fig) and was seen in seven sensitive proviruses and EN1-AN2 (Table in S1 Table). Of the four resistant plasma viruses with N137, 101603_030 and 101603_046, were neutralized by <1ug/ml of PGT121 and 101603_012 and 101603_017 required 6 and 12 ug/ml (Fig 2, Table in S1 Table). These four viruses had identical V1 sequence but exhibited sequence differences within the V2 insertion including positioning of PNGS.

Interestingly the glycan shifts we observed occurring at positions 337 and 339 in EN1 viruses were consistent with previous studies that have described shifts in PNGS affecting neutralization sensitivity and resistance [34]. Thus, it is likely that the polymorphisms we have encapsulated in our single time point sample represent common immune attack/viral escape strategies reflective of the on-going battle between the virus and the immune system. The location of PNGS in EN1 viruses was also analyzed using the recent glycan hole prediction software (https://www.hiv.lanl.gov/content/sequence/GLYSHIELDMAP/glyshieldmap.html) [87].

Absent gp120 PNGS or glycan holes occurred at positions 160, 234, 289, 295, 301, 339, and 392 in different viruses. The presence or absence of these PNGS was not consistently predictive of neutralization sensitivity or resistance of individual viruses. (Table in S1 Table).

### Expression and antibody binding to authentic and inferred EN1 envelope proteins

**Fig 5:**
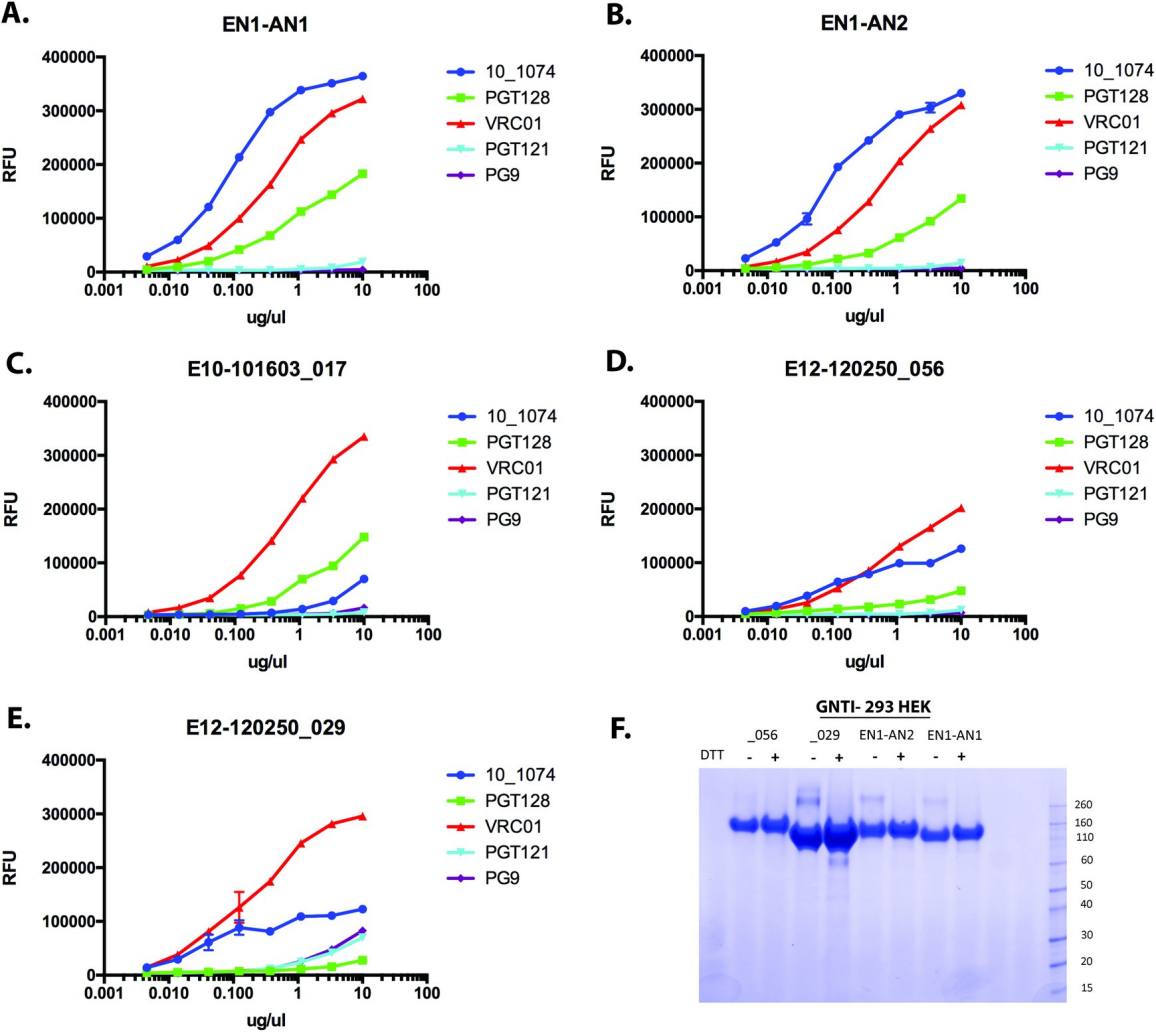
Comparison of bNAb binding to recombinant gp120 from native and inferred sequences. Sequences encoding gp120 from inferred envelope sequences EN1-AN1, EN1-AN2 (panels A and B) or authentic virus sequences (Panels C-E) were expressed in in GNTI- 293 HEK cells. The proteins were captured onto the wells of microtiter dishes and used in a fluorescence immunoassay (FIA) to measure the binding to a panel of prototypic bNAbs. These included bNAbs to the V1/V2 domain (PG9), the CD4 binding site (VRC01), or overlapping sites dependent on amino acids and glycans at the stem of the V3 domain (PGT121, PGT128, 10–1074). Panels EN1-AN1 and EN1-AN2 are sequences of inferred common ancestors occurring before the appearance of sequences resistant to neutralization by autologous plasma (see Fig 1). The E10-101603_17 sequence represents a plasma derived sequence resistant to neutralization by autologous plasma, whereas the E12-120250_56 sequence represents provirus derived sequence sensitive to neutralization by autologous plasma. The E12-120250_029 sequence represents the earliest provirus sequence in the phylogenetic tree (Fig 1) and was sensitive to neutralization by autologous plasma. Panel F, represents an SDS-PAGE gel of recombinant proteins from EN1 stained with SimpyBlue (ThermoFisher) before and after reduction with dithiothreitol (DTT). In this panel, _056 and -029 indicates protein from sequences EN12-120250_56 and E12-120250_29, respectively.

In order to investigate the antigenic structure of inferred Envs from EN1, we expressed inferred sequences encoding gp120s from the EN1-AN1 and EN1-AN2 constructs and three additional viruses representing different positions in the phylogenetic tree including EN10-10163-017 (a resistant plasma clone), and E12-120250_056 and E12-120250-029 (sensitive pro-virus clones) (Fig 1). The gp120 constructs were designed similarly to the ones used to produce the recombinant gp120s used for immunization in the 16,000 person RV144 HIV vaccine trial (see methods) [88, 89]. The genes were expressed in GnTI^-^ 293 HEK cells that limit N-linked glycosylation to mannose-5 structures required for the binding of several bN-mAbs [52, 60, 90, 91]. The proteins recovered from growth conditioned cell culture supernatants were then tested for binding by FIA to a panel of five representative bN-mAbs to the V1V2 domain(PG9), the V3 stem (10–1074, PGT121, PGT128) and the CD4 binding site (VRC01) (Fig 5). We found that all of the authentic proteins exhibited significant binding to the VRC01 bN-mAb, weak binding to 10–1074, and little if any binding to PGT121, PGT128 and PG9. In contrast, the two reconstructed ancestral Envs (EN1-AN1 and EN1-AN2) exhibited significant binding to VRC01 and 10–1074, and weak binding to PGT128. The inability to bind PG9 was surprising since pseudoviruses from these Envs were sensitive to neutralization by PG9 (Fig 2) and previous studies [60, 91] have showed that that other gp120s expressed in GnTI^-^ cells bound well to this antibody provided that they possessed PNGS at positions 156 and 160. The inability of these gp120s to bind PG9 might be attributable to the presence of glutamine (Q) at positon 170 rather than the usual lysine (K) at this position. Q at this position has been reported [37] to diminish neutralization by PG9, but the effect on bNAb binding has not previously been described. Interestingly both of the inferred ancestral Envs exhibited improved binding to the 10–1074 bn-mAb, a clonal relative to PGT121 [92]. This result suggests that these ancestral proteins possess a different antigenic structure than the three authentic viral proteins studied in this experiment.

## Discussion

The experiments described represent the first steps in the development of an improved HIV vaccine based on *envs* closely resembling those known to have previously elicited unusually broad and potent bNAbs in humans. The availability of these proteins will allow us to test the hypotheses that Envs from ENs have structural features that enhance the immunogenicity of epitopes recognized by bNAbs. This hypothesis could explain the surprising findings that properly folded trimeric envelope proteins, possessing virtually all of the epitopes recognized by bNAbs have, thus far, failed to consistently elicit bNAbs [15, 19–24]. Since bNAbs are not detected for several years post infection [29–32] and neutralization sensitive viruses appear to be cleared from circulation once bNAbs appear [29], the identification of envelopes that gave rise to bNAbs is challenging. This effort requires searching through the swarm of virus quasi-species that evolve in each individual EN in order to identify viruses with structures closely resembling those that elicited bNAbs. The present studies demonstrate that computational methods of sequence analysis, combined with virus neutralization data, can considerably narrow this search.

Several significant observations were made in these studies. First we found that proviral and plasma virus sequence data collected at a single time point could be combined with virus neutralization data to generate a phylogenetic tree that documents a series of naturally occurring mutations affecting neutralization sensitivity and resistance. This data also provided detailed molecular information with regard to shifts in the locations of PNGS and insertions in the V1/V2 and C3 domain that occur in the ongoing battle between the immune system and the virus. In particular, the pattern of PNGS involving positions 137, 337 and 339 were of the type that may affect the immunogenicity of bNAb epitopes. Second, we identified distinct structural features including a 9–14 amino acid insertion in the V2 domain and changes in the location of N-linked glycosylation sites in the V1 and V4 domains of gp120 that were significantly associated with the appearance of broad neutralization resistance. Third, using computational methods we identified fully functional Envs with sequences that appeared to be intermediates between the neutralization sensitive provirus sequences and the neutralization resistant plasma virus sequences and that possessed distinct antigenic structures as indicated by improved binding of bN-mAbs. Based on their occurrence immediately prior to the appearance of neutralization resistant plasma viruses, structural changes in regions known to be recognized by prototypic bN-mAbs, and differences in 10–1074 binding between earlier and later virus sequences, these intermediate sequences possess characteristics of the type that could have altered the antigenic structure and stimulated the formation of autologous bNAbs that drove the selection of neutralization resistant viruses.

It was surprising that such a complex picture of virus variation related to immune escape could be derived from data obtained at a single time point. Indeed the types of changes we observed (e.g. shifts in the locations of N-linked glycosylation sites, insertions in the V1/V2 domain) have primarily been previously described in studies of longitudinal specimens [34, 41, 43]. We found it interesting that a neutralization sensitive sequence (EN1-AN2) defined the base of the resistant clade. EN1-AN2 clearly has the epitopes required for sensitivity to autologous neutralization and lacked the neutralization escape mutations that evolved in EN1. The result supports our choice of this node as the closest common ancestor of neutralization resistant viruses and suggests that the methodology was sound.

In large part we attribute the success of this analysis to the Monogram Phenosense and Trofile assay methods that pre-selects for functional *envs* and eliminates the defective proviral *envs* found in PBMCs. Although we seem to have reconstructed *env* sequences close to the time that viruses resistant to bN-mAbs evolved, it was unlikely that this “proof of concept” approach captured the entire history of virus evolution with the relatively small number of sequences analyzed. Undoubtedly, analysis of larger numbers of sequences will provide a more detailed picture of virus evolution in this individual. Indeed we found that that the epitopes recognized by several known bN-mAbs (e.g., PG9, PG16, PGT121) were present even in the earliest viruses in the phylogenetic tree (proviral population). Previous longitudinal studies have reported that glycan dependent epitopes recognized by bN-mAbs are not always present in early infections and appear to evolve in response to selective pressure mediated by strain specific neutralizing antibodies [93]. Thus, future studies involving the selection of greater numbers of sequences and characterization of neutralization sensitivity with larger panel of bN-mAbs, will undoubtedly add to our understanding of the evolution of viral sequences in this individual and the identification of additional intermediate sequences.

### What is the rate limiting step in the formation of bNAbs?

Multiple studies have reported that bNAbs do not typically appear in HIV infected individuals until 2–3 years post infection [29–32]. The critical issue for vaccine development is whether this delay is attributable to a prolonged clonal selection and evolution of immunoglobulin genes with the physical characteristics of bNAbs such as long CDR3 domains and hyper-mutated immunoglobulin variable regions [93, 94], or to a prolonged evolution of *env* sequences with rare structural features required to enhance the immunogenicity of epitopes recognized by bNAbs. While our data does not allow us to distinguish between these two hypotheses, the envelopes we have isolated provide the basis for further experiments that might resolve this issue. First we can directly compare the immunogenicity of the reconstructed Env proteins (monomers and trimers) to earlier sensitive proviral Envs. Second, we can measure the extent to which the ancestral Envs bind the germline antibody genes [95] representative of the five different specificities of bNAbs compared to authentic Envs throughout the EN1 phylogeny. Third, the Envs we have isolated can be used in guided immunization strategies [4, 5, 94]. All of these approaches should be informative. However the use of envelope proteins known to have elicited bNAbs in humans, such as those described in this paper, may be advantageous compared to envelope proteins included in previous candidate HIV vaccines that were selected without regard to the immune response in the host.

## Supporting information

S1 Fig **A-I. Plots comparing serum and monoclonal antibody neutralization titers of EN1 viruses with and without select features listed in Table 5.** The virus serum and antibody neutralization data from Table 5 were plotted as a function of the presence or absence of the listed feature. The distributions were compared by unpaired, nonparametric Mann Whitney tests. *P* values for differences in neutralization titers are indicated.
(DOCX)

S1 Table Listing of PNGS present in EN1 viruses and reconstructions encompassing the V1/V2; C2/V3/C3) and C4 domains (HXB2 numbering). (DOCX)
