# Supplementary figures and images for: Ancestral sequences from an elite neutralizer proximal to the development of neutralization resistance as a potential source of HIV vaccine immunogens
Source: PLoS One. 2019 Apr 10;14(4):e0213409. doi: 10.1371/journal.pone.0213409 (PMC6457492; doi:10.1371/journal.pone.0213409)

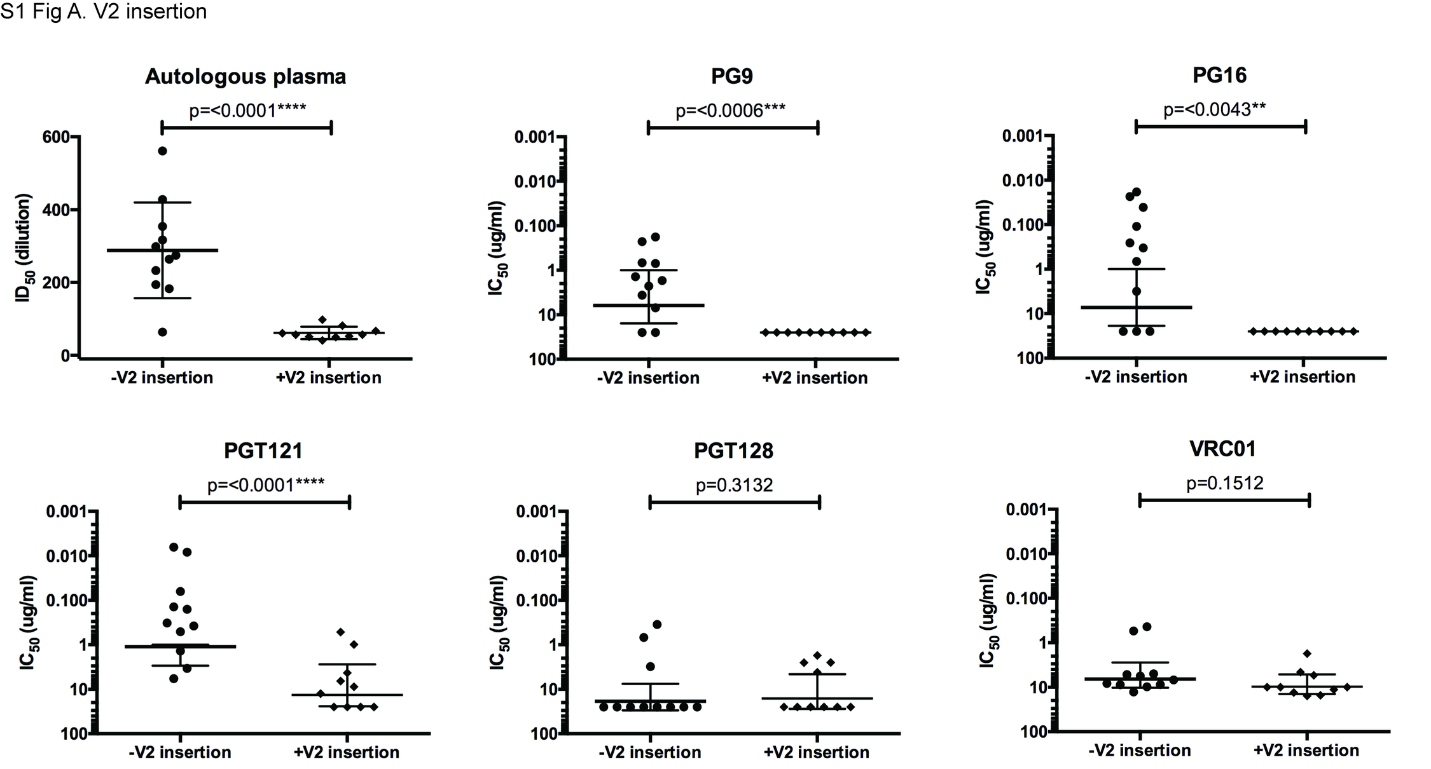


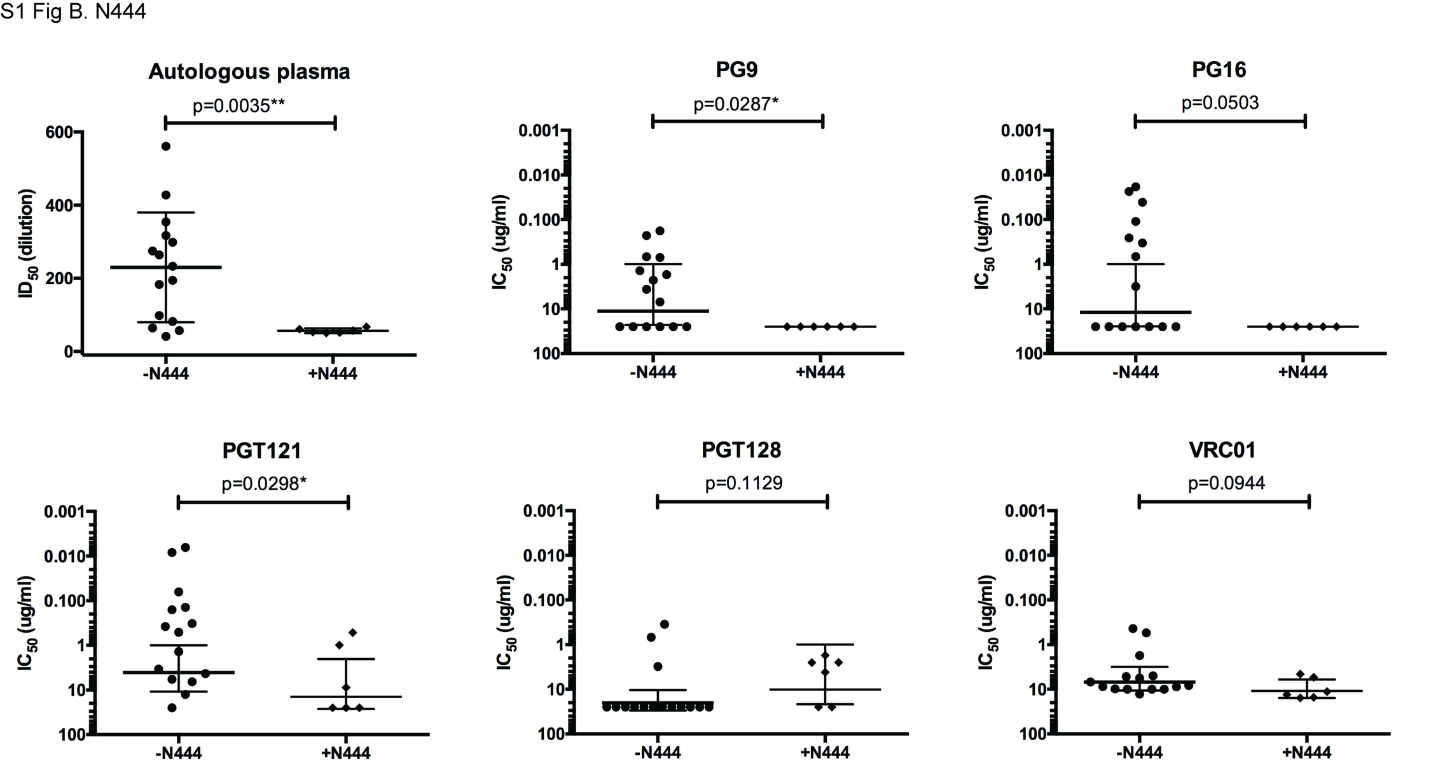


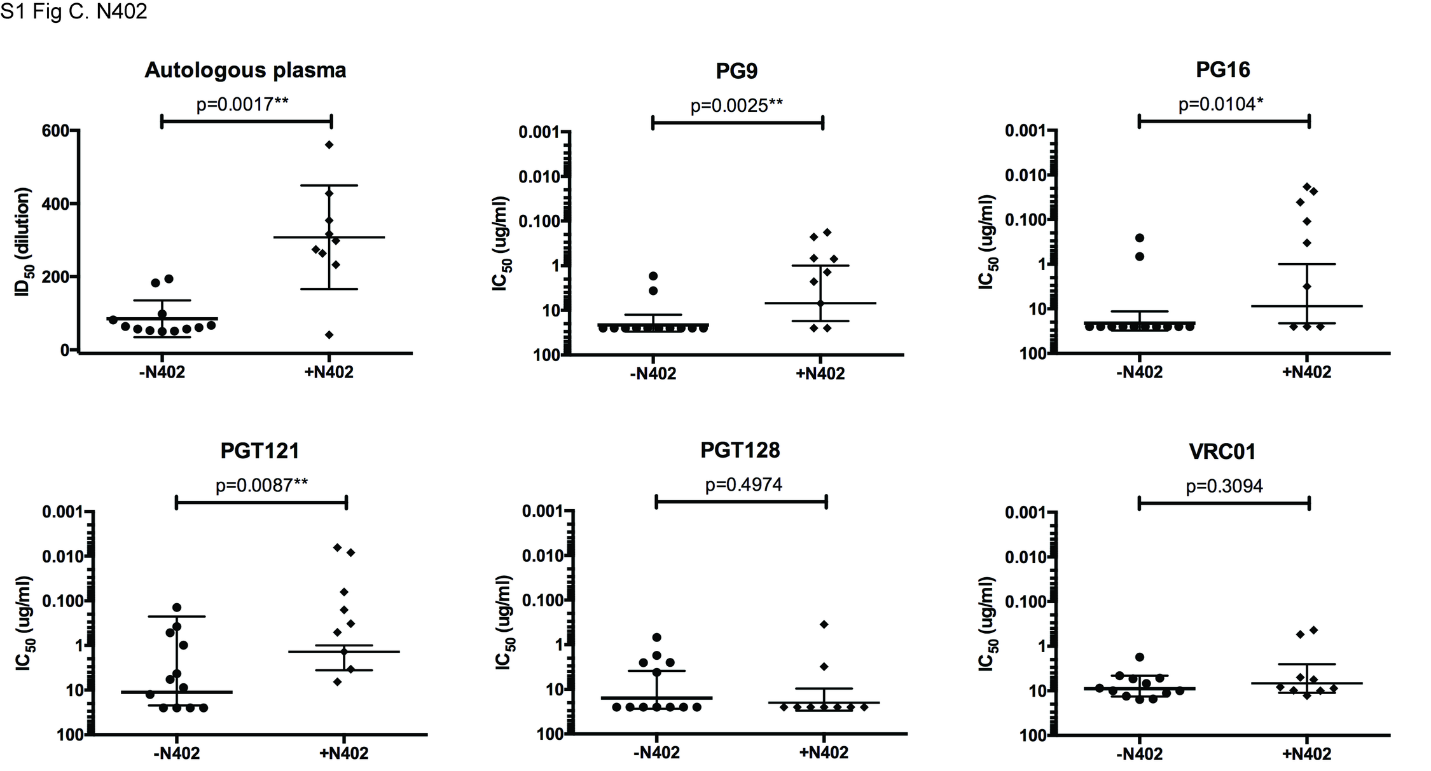


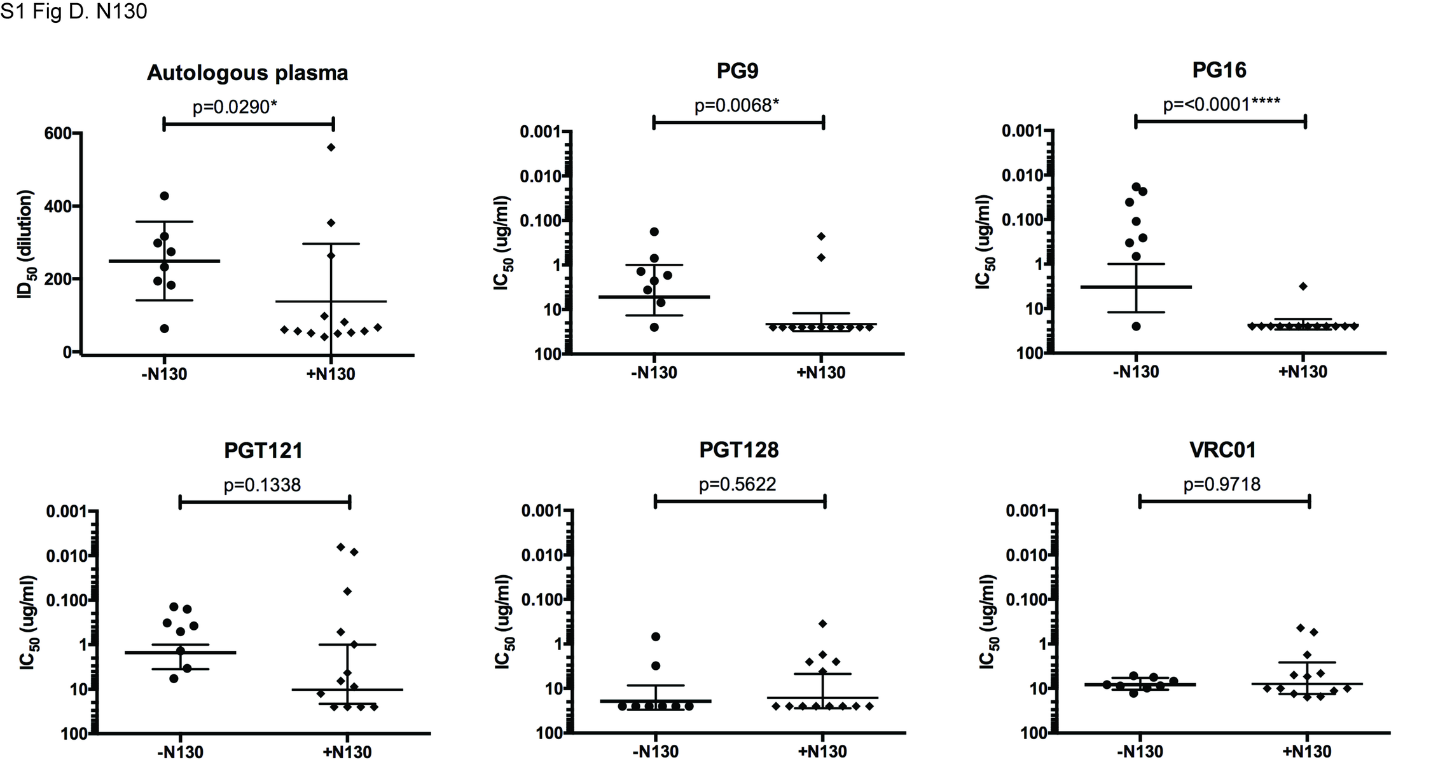


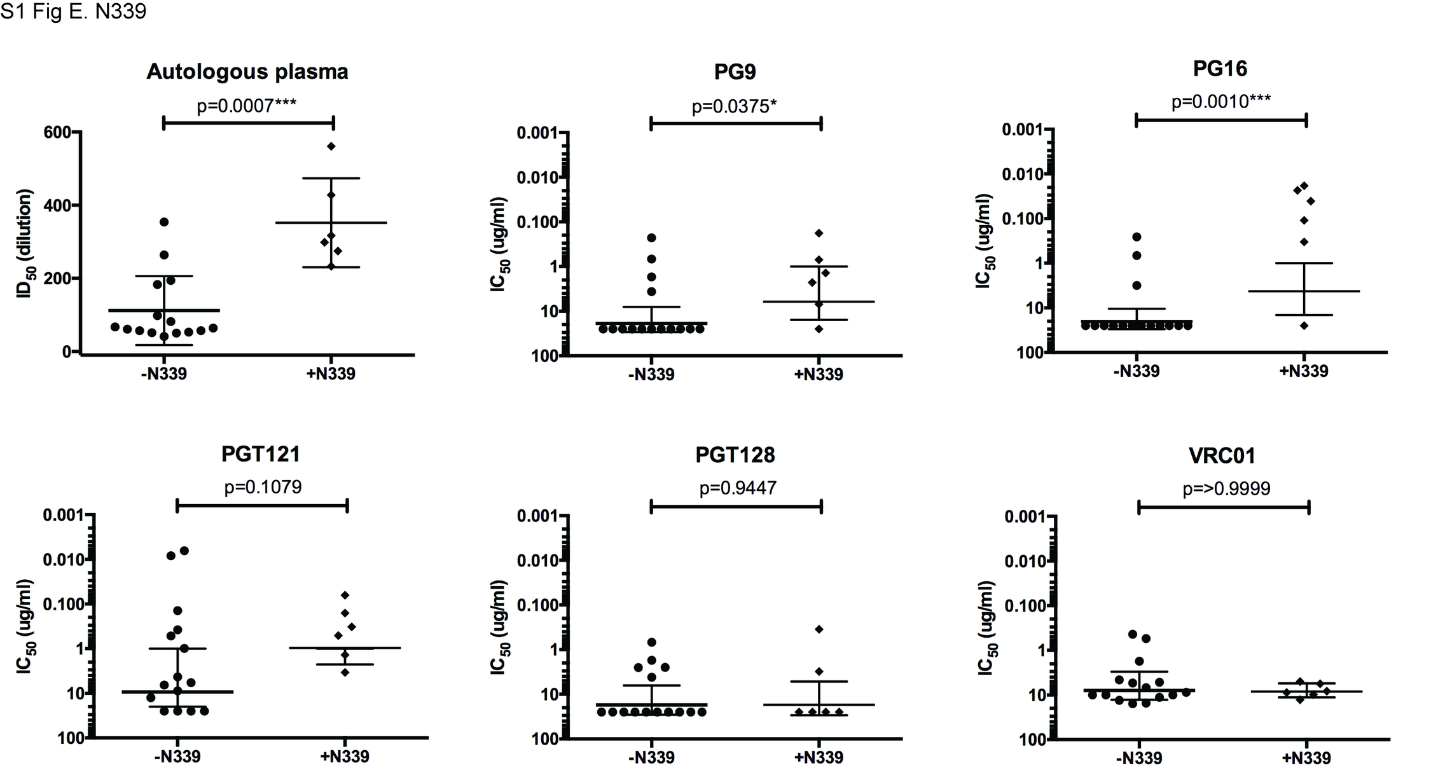


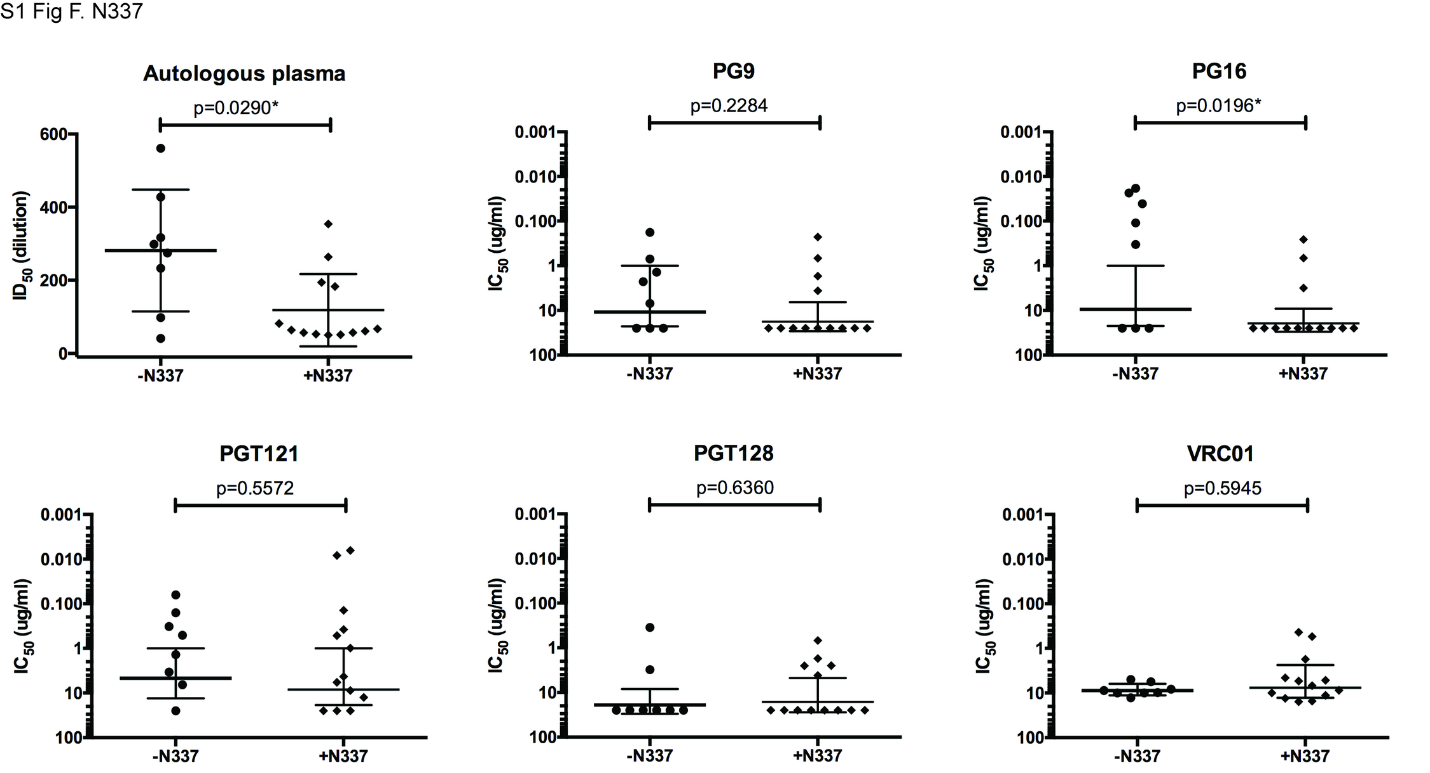


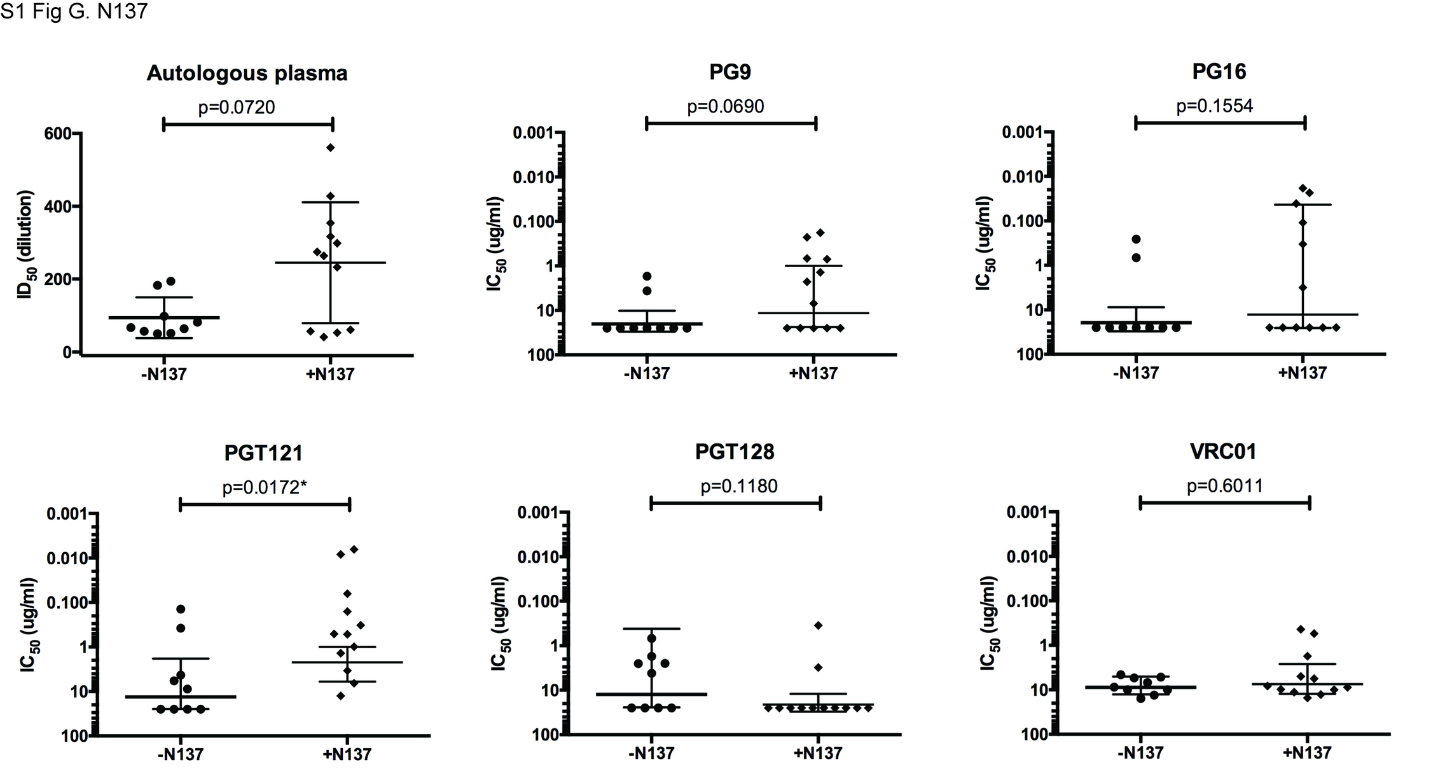


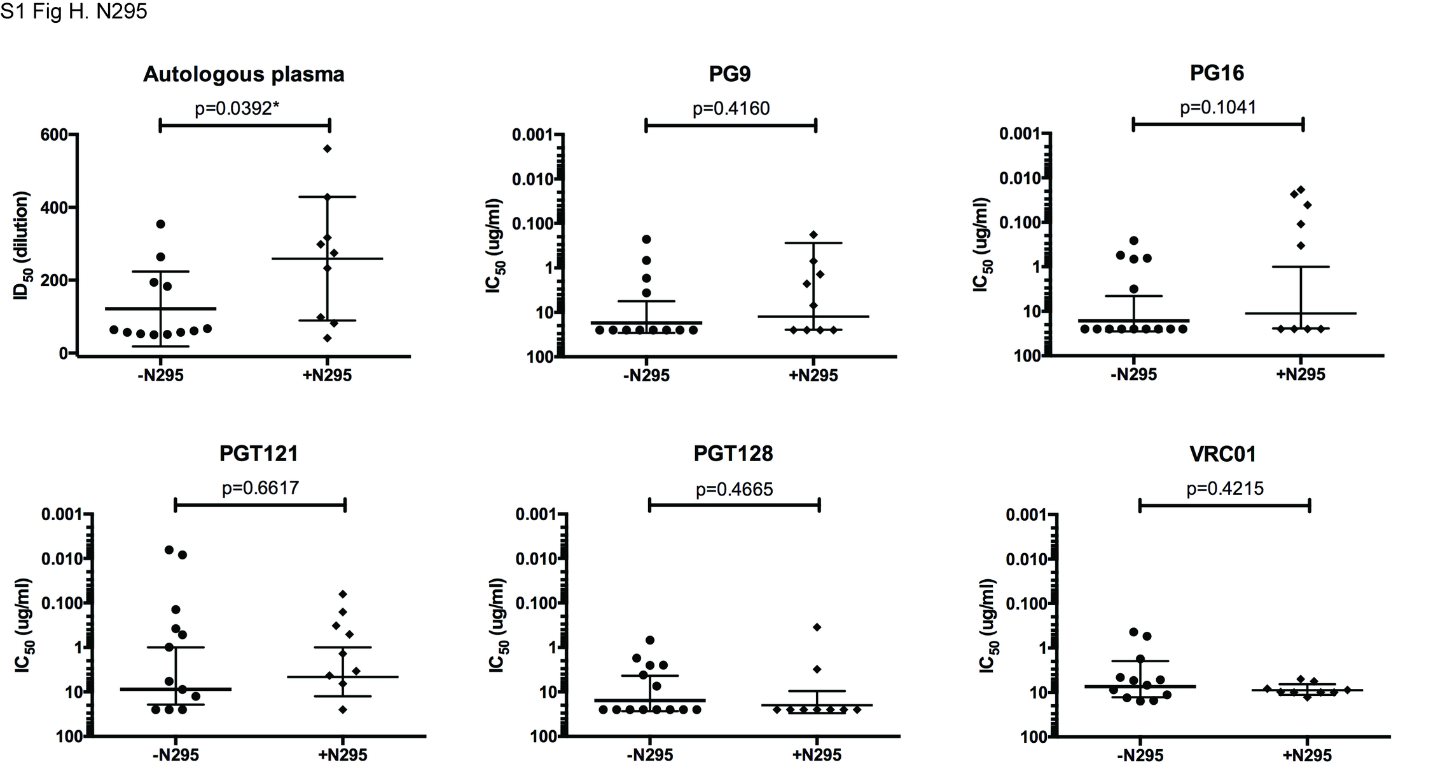


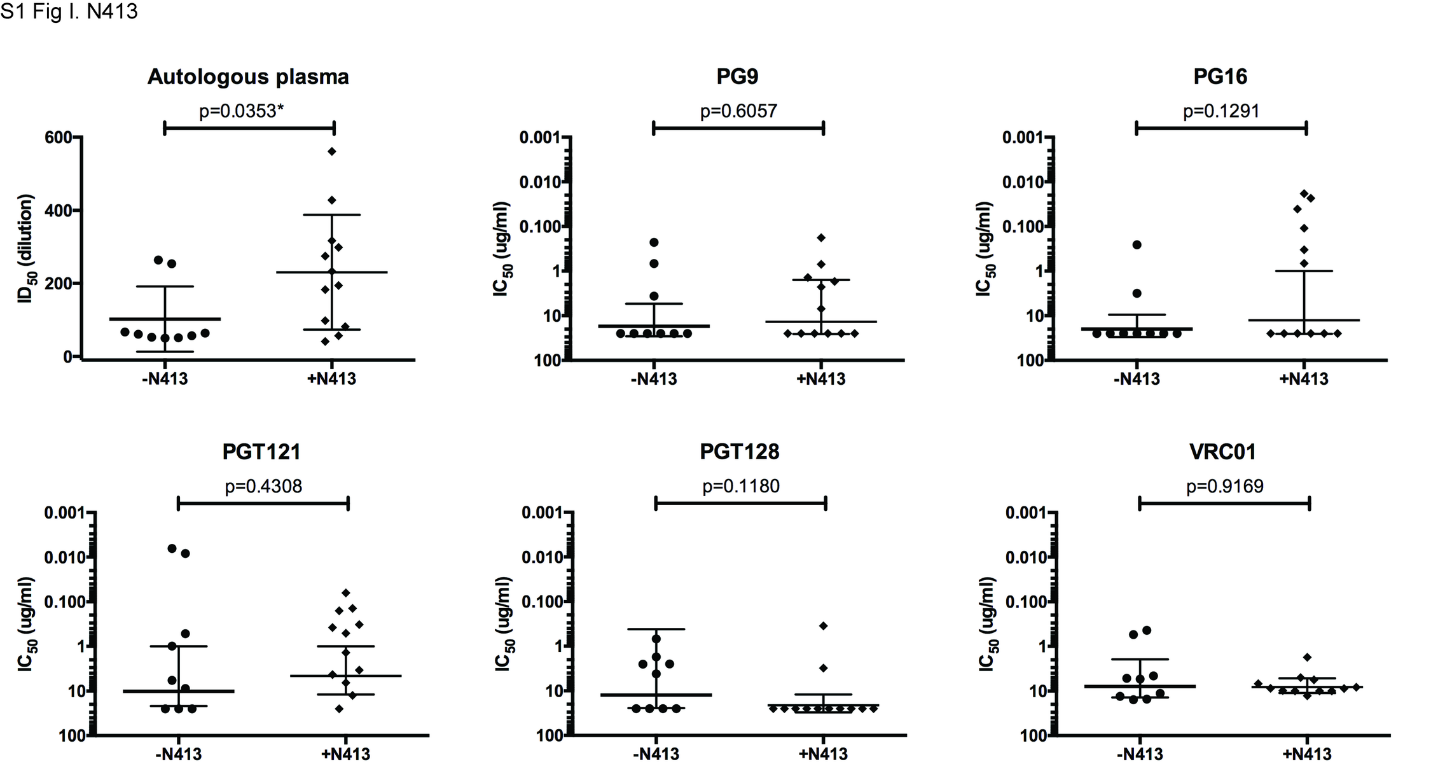

Supplement: S1 Fig — A-I. Plots comparing serum and monoclonal antibody neutralization titers of EN1 viruses with and without select features listed in Table 5. The virus serum and antibody neutralization data from Table 5 were plotted as a function of the presence or absence of the listed feature. The distributions were compared by unpaired, nonparametric Mann Whitney tests. P values for differences in neutralization titers are indicated. (DOCX) [file pone.0213409.s001.docx]
