# Supplementary material for: Ancestral sequences from an elite neutralizer proximal to the development of neutralization resistance as a potential source of HIV vaccine immunogens
Source: PLoS One. 2019 Apr 10;14(4):e0213409. doi: 10.1371/journal.pone.0213409 (PMC6457492; doi:10.1371/journal.pone.0213409)
Supplement: S1 Table — (DOCX) [file pone.0213409.s002.docx]

**S1 Table. Listing of PNGS present in EN1 viruses and reconstructions encompassing the V1/V2; C2/V3/C3) and C4 domains (HXB2 numbering)**.**

| Virus | SNT | V1 | | | | | | | | V2 | | | | No. V1V2 PNGS |
| --- | --- | --- | --- | --- | --- | --- | --- | --- | --- | --- | --- | --- | --- | --- |
|  |  | 130 | 133 | 135 | 137 | 140 | 142 | 145+ | 149 | 156 | 160 | 184+ | 188 |  |
| E10-101603_014 | 51 | + | - | + | - | + | - | - | + | + | + | ++ | + | 9 |
| E10-101603_022 | 50 | + | - | + | - | + | - | - | + | + | + | ++ | + | 9 |
| E10-101603_026 | 57 | + | - | + | - | + | - | - | + | + | + | ++ | + | 9 |
| E10-101603_047 | 67 | + | - | + | - | + | - | - | + | + | + | ++ | + | 9 |
| E10-101603_030 | 61 | + | - | - | + | - | + | - | + | + | + | ++ | - | 8 |
| E10-101603_046 | 53 | + | - | - | + | - | + | - | + | + | + | ++ | - | 8 |
| E10-101603_012 | 41 | + | - | - | + | - | + | - | + | + | + | ++ | - | 8 |
| E10-101603_017 | 57 | + | - | - | + | - | + | - | + | + | + | ++ | + | 9 |
| E10-101603_039 | 64 | - | - | + | - | + | - | - | + | + | + | + | - | 6 |
| E12-120250_018 | 82 | + | - | + | - | + | - | ++ | + | + | + | + | + | 10 |
| E12-120250_076 | 98 | + | - | + | - | + | - | ++ | + | + | + | + | - | 9 |
| E12-120250_006 | 183 | - | + | - | - | - | + | - | + | + | + | - | - | 5 |
| E12-120250_011 | 194 | - | + | - | - | - | + | - | + | + | + | - | - | 5 |
| E12-120250_005 | 275 | - | - | - | + | - | + | - | + | + | + | + | - | 6 |
| E12-120250_095 | 299 | - | - | - | + | - | + | - | + | + | + | + | - | 6 |
| E12-120250_079 | 317 | - | + | - | + | - | + | + | + | + | + | - | + | 8 |
| E12-120250_052 | 233 | - | + | - | + | - | + | + | + | + | + | - | + | 8 |
| E12-120250_056 | 428 | - | + | - | + | - | + | + | + | + | + | - | + | 8 |
| E12-120250_040 | 561 | + | - | - | + | - | + | + | + | + | - | - | + | 7 |
| E10-101603_029 | 264 | + | - | - | + | - | - | - | + | + | + | - | + | 6 |
| E12-120250_029 | 354 | + | - | - | + | - | - | + | + | + | + | - | + | 7 |
|  |  |  |  |  |  |  |  |  |  |  |  |  |  |  |
| EN1-AN1 | 204 | - | + | - | - | - | + | - | + | + | + | - | - | 5 |
| EN1-AN2 | 457 | + | - | - | + | - | + | - | + | + | + | + | + | 8 |

| Virus | SNT | C2 | | | | | | | V3 | C3 | | | | | V4 | | | | | | | C4 | | No. C2-C4 PNGS |
| --- | --- | --- | --- | --- | --- | --- | --- | --- | --- | --- | --- | --- | --- | --- | --- | --- | --- | --- | --- | --- | --- | --- | --- | --- |
|  |  | 197 | 234 | 241 | 262 | 276 | 289 | 295 | 301 | 332 | 337 | 339 | 356 | 362 | 386 | 392 | 394 | 396+ | 402 | 406 | 413 | 444 | 448 |  |
| E10-101603_014 | 51 | + | + | + | + | + | - | - | + | + | + | - | + | + | + | + | - | - | - | + | - | + | + | 15 |
| E10-101603_022 | 50 | + | + | + | + | + | - | - | + | + | + | - | + | + | + | + | - | - | - | + | - | + | + | 15 |
| E10-101603_026 | 57 | + | + | + | + | + | - | - | + | + | + | - | + | + | + | + | - | - | - | + | - | + | + | 15 |
| E10-101603_047 | 67 | + | + | + | + | + | - | - | + | + | + | - | + | + | + | + | - | - | - | + | - | + | + | 15 |
| E10-101603_030 | 61 | + | + | + | + | + | - | - | + | + | + | - | + | + | + | + | - | - | - | + | - | + | + | 15 |
| E10-101603_046 | 53 | + | + | + | + | + | - | - | + | + | + | - | + | + | + | + | - | - | - | + | - | + | + | 15 |
| E10-101603_012 | 41 | + | + | + | + | + | - | + | + | + | - | - | + | + | + | - | - | + | + | + | + | - | + | 16 |
| E10-101603_017 | 57 | + | + | + | + | + | - | - | + | + | + | - | + | - | + | - | - | + | - | + | + | - | + | 14 |
| E10-101603_039 | 64 | + | + | + | + | + | - | - | + | + | + | - | + | - | + | + | - | + | - | + | - | - | + | 14 |
| E12-120250_018 | 82 | + | + | + | + | + | - | + | + | + | + | - | + | - | - | - | - | + | - | + | + | - | + | 14 |
| E12-120250_076 | 98 | + | + | + | + | + | - | + | + | + | - | - | + | - | - | - | - | + | - | + | + | - | + | 13 |
| E12-120250_006 | 183 | + | + | + | + | + | - | - | + | + | + | - | + | - | + | - | - | + | - | + | + | - | + | 14 |
| E12-120250_011 | 194 | + | + | + | + | + | - | - | + | + | + | - | + | - | + | - | - | + | - | + | + | - | + | 14 |
| E12-120250_005 | 275 | + | + | + | + | + | - | + | + | + | - | + | + | - | + | + | - | + | + | + | + | - | + | 17 |
| E12-120250_095 | 299 | + | + | + | + | + | - | + | + | + | - | + | + | - | + | + | - | + | + | + | + | - | + | 17 |
| E12-120250_079 | 317 | + | - | + | + | + | - | + | + | + | - | + | + | - | + | - | + | + | + | + | + | - | + | 16 |
| E12-120250_052 | 233 | + | - | + | + | + | - | + | + | + | - | + | + | - | + | - | + | + | + | + | + | - | + | 16 |
| E12-120250_056 | 428 | + | - | + | + | + | - | + | + | + | - | + | + | - | + | - | + | + | + | + | + | - | + | 16 |
| E12-120250_040 | 561 | + | - | + | + | + | + | + | + | + | - | + | + | - | + | - | - | + | + | + | + | - | + | 16 |
| E10-101603_029 | 264 | + | - | + | + | + | + | - | - | + | + | - | + | + | + | + | - | - | + | + | - | - | + | 14 |
| E12_120250_029 | 354 | + | - | + | + | + | + | - | - | + | + | - | + | + | + | + | - | - | + | + | - | - | + | 14 |
|  |  |  |  |  |  |  |  |  |  |  |  |  |  |  |  |  |  |  |  |  |  |  |  |  |
| EN1-AN1 | 204 | + | + | + | + | + | - | - | + | + | + | - | + | - | + | - | - | + | - | + | + | - | + | 14 |
| EN1-AN2 | 457 | + | + | + | + | + | - | - | + | + | + | - | + | - | + | - | - | + | - | + | + | - | + | 14 |

** Plus (+) indicates presence of a PNGS, Minus (-) indicates absence of a PNGS. Viruses are listed in phylogenetic order. Proviruses are shaded. Autologous sera neutralization titers (SNT) are 1/dilution**.**
